# Principles and Applications of Vibrational Spectroscopic Imaging in Plant Science: A Review

**DOI:** 10.3389/fpls.2020.01226

**Published:** 2020-08-07

**Authors:** Krzysztof B. Beć, Justyna Grabska, Günther K. Bonn, Michael Popp, Christian W. Huck

**Affiliations:** ^1^ CCB-Center for Chemistry and Biomedicine, Institute of Analytical Chemistry and Radiochemistry, Leopold-Franzens University, Innsbruck, Austria; ^2^ ADSI, Austrian Drug Screening Institute, Innsbruck, Austria; ^3^ Michael Popp Research Institute for New Phyto Entities, University of Innsbruck, Innsbruck, Austria

**Keywords:** hyperspectral, multispectral, imaging, near-infrared, FT-IR, Raman, plant, vibrational spectroscopy

## Abstract

Detailed knowledge about plant chemical constituents and their distributions from organ level to sub-cellular level is of critical interest to basic and applied sciences. Spectral imaging techniques offer unparalleled advantages in that regard. The core advantage of these technologies is that they acquire spatially distributed semi-quantitative information of high specificity towards chemical constituents of plants. This forms invaluable asset in the studies on plant biochemical and structural features. In certain applications, non-invasive analysis is possible. The information harvested through spectral imaging can be used for exploration of plant biochemistry, physiology, metabolism, classification, and phenotyping among others, with significant gains for basic and applied research. This article aims to present a general perspective about vibrational spectral imaging/micro-spectroscopy in the context of plant research. Within the scope of this review are infrared (IR), near-infrared (NIR) and Raman imaging techniques. To better expose the potential and limitations of these techniques, fluorescence imaging is briefly overviewed as a method relatively less flexible but particularly powerful for the investigation of photosynthesis. Included is a brief introduction to the physical, instrumental, and data-analytical background essential for the applications of imaging techniques. The applications are discussed on the basis of recent literature.

## Introduction

From the point-of-view of physicochemical methods of analysis, plants form a challenging subject. As complex, microstructured forms with multi-constituent chemical composition, their comprehensive studies require sensitive and chemically selective methods. On the other hand, conventional sample preparations may result in non-representative results, e.g., chemical clearing or drying as prerequisites for plant sample interrogation. It is preferable to retain the capability of examining living forms in their native state. Having these general remarks in mind, vibrational spectral imaging techniques offer a superb potential. These methods have high chemical specificity, enabling them to construct a chemical image of the sample, within which the distribution of compounds is available even at cellular and sub-cellular level. This may be accomplished in a non-destructive way, and in certain cases with no sample preparation. In numerous cases, examinations *in vivo* are feasible. In other cases, fractions can be isolated from the plant material for further spectroscopic analysis. Both pathways are suitable for obtaining spectral images that feature characteristic key bands of individual components. These bands yield information on the chemical composition of the plant sample, e.g., structural constituents, or primary and secondary metabolites. The identified chemicals may be used as the markers, further interpreted to discriminate different species, or chemotypes among the same species. Insights into plant microstructures, physiology and biochemistry are available. With use of data-analytical methods, information on various properties of a plant sample may be unraveled and presented in the form of an easily accessible image; often, quantitative or semi-quantitative data on the chemical content may be obtained. These advantages have been well recognized in plant science, with spectral imaging becoming an increasingly popular investigation tool (Elsayad, 2019).

The present review aims to overview vibrational spectral imaging in the field of plant-related research. The methods in scope include infrared (IR), near-infrared (NIR) and Raman imaging (Schulz et al., 2014). Additionally, fluorescence imaging is briefly overviewed; it is based on a different physical principle, yet gained profound use in plant science, e.g., in the studies of photosynthesis. As it delivers information of complementary character, it seems advantageous to compare fluorescence and vibrational imaging, with aim to better expose the advantages and limitations of each of these techniques. The purpose of this work is to dissect the methods and applications in an interpretative way, while assessing their relative usefulness at various directions of research and analysis, in which plant-related samples are the common denominator. Brief introductions to the background phenomena, instrumentation, image generation and data analytical methods, spectra interpretation, and related information are included, while the interested reader is pointed to the referenced literature for more exhaustive information. The discussion of these fundamental topics is directed towards the better understanding of the final performance and applicability of reviewed techniques in plant science. The majority of reviewed applications are based on literature published over the past few years, with selected exceptions that present relevant information or have initiated significant lanes of research.

## Fundamentals and Principles of Vibrational Spectroscopy and Imaging

### Basic Information Related to Spectra Origin and Interpretation

In vibrational (e.g., infrared, IR; near-infrared, NIR; Raman) spectroscopy the interaction with electromagnetic radiation triggers the irradiated molecules between their quantum vibrational states *v*. The vibrational (i.e., internal) degrees of freedom of molecules correspond to oscillating changes in bond lengths and angles between these bonds, or in other words stretching and deformation vibrations (modes), respectively. In Raman and IR spectra, the most meaningful are bands resulting from fundamental transitions, in contrast NIR spectra re populated by overtones and combination bands. IR and NIR are absorption spectroscopies, the appearance of a band in the corresponding spectra results from an act of photon absorption. In contrast, Raman spectroscopy probes the vibrational excitations of molecules, although the mechanisms of the interaction with electromagnetic radiation is entirely different (Hammes, 2005). It involves Raman effect, which is an inelastic scattering of photons (Gardiner and Graves, 1989; Hammes, 2005). The symmetry of some vibrations prevents the absorption of photon, leading to so-called (either IR- or Raman-) inactive modes. The probability of absorption, connected with the band intensity (i.e., spectral intensity), is directly proportional to the extent of the dipole moment change, which is the selection rule in this case. Therefore, IR and NIR spectroscopies are particularly sensitive toward the vibrations of polar functional groups. Raman band intensities are dictated by different criteria (Gardiner and Graves, 1989; Hammes, 2005). Raman effect is intrinsically weak, therefore, in order to measure a useful Raman signal, a relatively strong source of monochromatic light is necessary to yield high amount of incident photons. Practical considerations make laser emitting in the visible, near-infrared, or visible/near-ultraviolet range most useful in this role. However, such source is prone to stimulate fluorescence in certain types of samples. This occurs in chlorophyll-rich samples, e.g., plant tissues and related materials. In contrast, fluorescence is a form of luminescence, i.e., spontaneous emission of radiation by a fluorescent molecule (fluorophore) after photon-induced excitation. The spectroscopy based on this phenomenon (fluorimetry or spectrofluorometry) probes electronic and vibrational energy levels of molecules (Lichtman and Conchello, 2005; Kokawa et al., 2015). To stimulate fluorescence, the fluorophore first needs to be electronically excited, e.g., by using UV radiation with wavelengths matching the electronic transitions of the fluorophore. The fluorescent response depends on and is specific to both the electronic and vibrational structure of the fluorophore. Numerous biomolecules found in plants are strong fluorophores, e.g., the ubiquitous chlorophyll or various bio-active compounds found in medicinal plants such as quinine with maximum fluorescence in Vis region.

IR and Raman spectra feature high level of chemical specificity, as the spectral bands are relatively sharp and their positions and intensities can be reliably ascribed to the specific chemical functional groups. This enables to identify the chemical compounds typically present in plant samples (**Table 1**). Exhaustive correlation tables for specific IR and Raman peaks relevant to plant-related samples can be found in literature, e.g., in Schulz and Barańska (2007), or Lin-Vien et al. (1991). NIR spectra preserve to an extent this advantageous feature with absorption bands appearing at predictable wavenumbers (**Table 2**). Yet, in practice, one encounters several effects that prevent straightforward interpretation of NIR spectra. Detailed discussions of this topic can be found in literature (Ozaki et al., 2018; Beć and Huck, 2019). In sharp contrast, fluorescence spectra depend on the excitation and emission properties of fluorophores and are generally complex with overlapped band structures forming several distinct intensity maxima (Albani, 2004). Fluorescence profile also depends on the matrix properties and the rigidity of the medium and the measurement conditions as well. For those reasons, the approach to the interpretation of fluorescence is much less systematic than in the previously introduced techniques (Albani, 2004).

**Table 1:** Characteristic fundamental bands in infrared (IR) and Raman spectra of chemical compounds commonly found in plants.

Wavenumber in cm^-1^	Vibrational mode assignment and the associated most characteristic compounds
3,500–3,200	O-H and N-H stretch: carbohydrates, proteins, alcohols and phenolic compounds
2,960–2,950	CH_3_ asymmetric stretching: mainly lipid with a little contribution from protein, carbohydrate, and nucleic acid
2,930–2,920	CH_2_ asymmetric stretching: mainly lipid with a little contribution from protein, carbohydrate, and nucleic acid
2,875–2,870	CH_3_ symmetric stretching: mainly proteins with a little contributions from lipid, carbohydrate, and nucleic acid
2,860–2,840	CH_2_ symmetric stretching: mainly lipids with a little contributions from protein, carbohydrate, and nucleic acid
1,745–1,730	Saturated ester C=O stretch: phospholipid, cholesterol, ester, hemicellulose, pectin, lignin, suberin/cutin esters
1,650–1,630	Amide I (C=O stretch): protein, pectin, water associated cellulose or lignin, alkaloids
1,630–1,620	C=C stretch: phenolic compound
1,610–1,590	C=O aromatic stretch: lignin, alkaloid
1,560–1,540	Amide II (C=N and N-H stretch): mainly protein
1,515–1,505	C=C aromatic stretch: lignin
1,460–1,455	Amide III (aromatic hydrocarbons): mainly protein
1,455–1,440	C-H asym bending of CH_2_ and CH_3_: cell wall polysaccharide, lipid and protein
1,430–1,420	O-H bend: cell wall polysaccharide, alcohol and carboxylic acid
1,380–1,370	C–H sym bending of CH_2_ and CH_3_: cell wall polysaccharide, lipid and protein
1,375–1,365	C–H bend: cellulose and hemicellulose
1,250–1,240	C=O stretch: pectic substances, lignin, hemicellulose, suberin/cutin esters
1,235	Amide IV (C=N and N–H stretching): mainly protein
1,235–1,230	C–O stretch: lignin, xylan
1,205–1,200	O–H in plane bend: cellulose
1,170–1,160	C–O–C asym stretch: cutin
1,160–1,150	Symmetric bonding of aliphatic CH_2_, OH, or C–O stretch of various groups: cell wall polysaccaride
1,145–1,140	C–O–C asym stretch: cellulose (β-1.4 glucan)
1,110–1,105	C–O–C sym stretch: cutin
1,105–1,100	Antisymmetric in-phase: pectic substance
1,085–1,075	C–O deformation: secondary alcohol, aliphatic ester
1,075–1,070	C–O ring stretch: rhamnogalactorunan, b-galactan
1,065–1,060	C–O stretch: cell wall polysaccarides (glucomannan)
1,045–1,030	O–H and C–OH stretch: cell wall polysaccarides (arabinan, cellulose)
990–980	C–O stretch: cutin
900–890	C–H deformation: arabinan
895–890	C–O valence vibration: galactan
875–870	C–O stretch: β–D-fructose

Adapted from Türker-Kaya and Huck (2017) under CC-BY 4.0 license.

**Table 2:** Characteristic near-infrared (NIR) bands of chemical compounds commonly found in plants.

Wavenumber in cm^-1^	Wavelength in nm	Vibrational mode assignment and the associated most characteristic compounds
8,251	1,212	3 C–H str.: carbohydrates
7,375	1,356	2 C–H str. + C–H def.: carbohydrates
7,168	1,395	2 C–H str. + C–H def.: carbohydrates
6,983	1,432	2 N–H str.: proteins
6,748	1,482	2 O–H str.: carbohydrates
6,662	1,501	2 N–H str.: carbohydrates
6,494	1,540	2 O–H str. (intermol. H-bond): starch
6,394	1,564	2 N–H str.: proteins
6,196	1,614	2 C–H str.: carbohydrates
6,053	1,652	2 C–H str.: carbohydrates
5,896	1,696	2 C–H str.: carbohydrates
5,627	1,777	2 C–H str.: plant fiber composed of cellulose, lignin and other carbohydrates
5,507	1,816	O–H str. + 2 C–O str.: plant fiber composed of cellulose, lignin and other carbohydrates
5,120	1,953	3 C–O str.: carbohydrates
4,878	2,050	N–H sym. str. + amide II: proteins
4,824	2,073	O–H str. + O–H def.: alcohols
4,643	2,154	Amide I + amide III: proteins
4,439	2,253	O–H str. + O–H def.: starch
4,363	2,292	N–H str. + CO str.: proteins

Adapted from Türker-Kaya and Huck (2017) under CC-BY 4.0 license.

### Techniques of Spectroscopic Imaging and Microspectroscopy

#### Basics of the Spectral Image Acquisition

**Figure 1:**
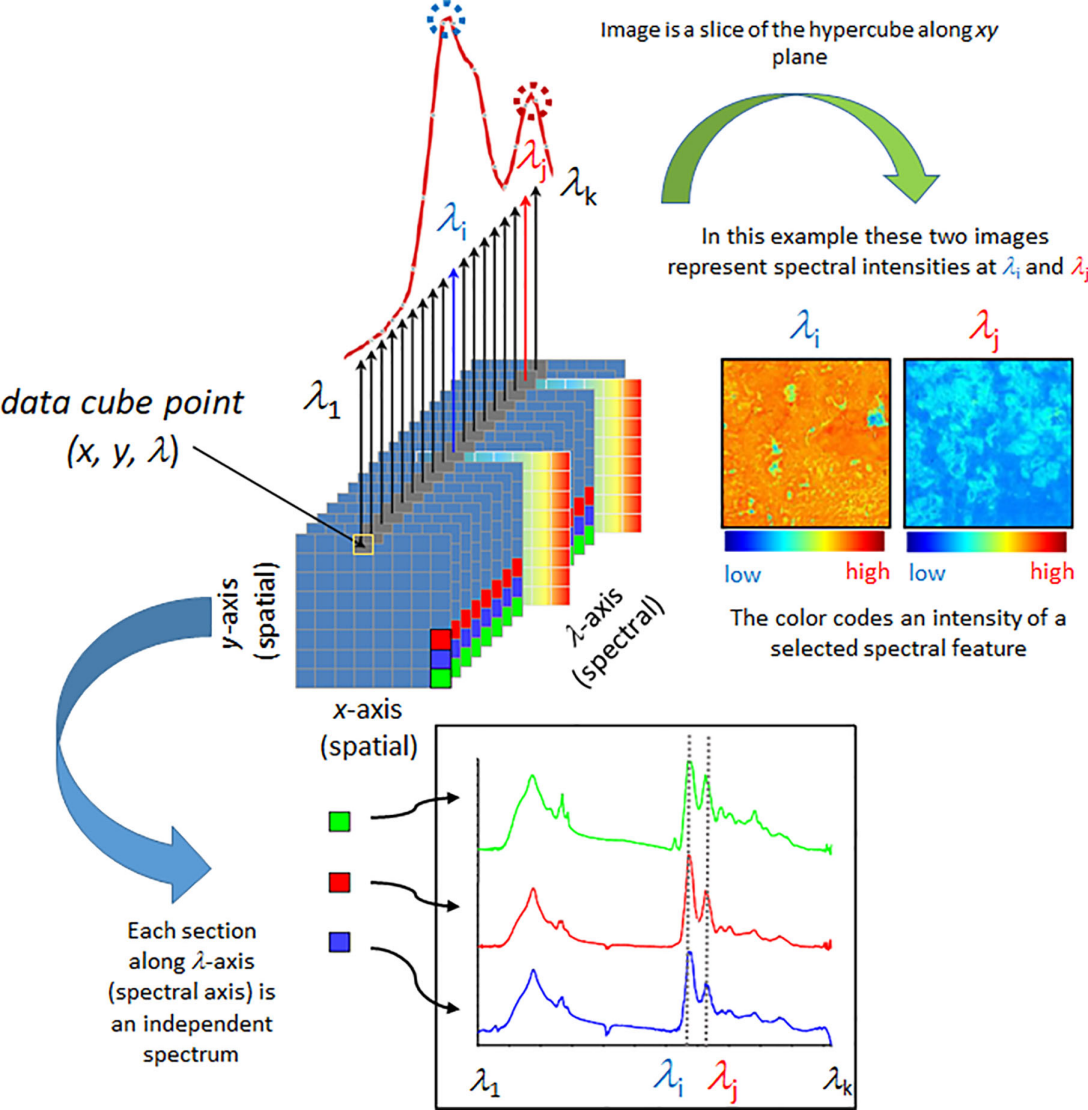
Simplified scheme presenting fundamental principles of a spectral data hypercube and visualization based on the most straightforward spectral information available, spectral intensity. Reproduced with permission from Elsevier (Beć et al., 2020).

Spectral imaging is a spatially resolved technique able to acquire the spectrum from a specified point at the sample surface. In addition to the spectral wavelength-dependent (*Λ*) also spatial (*x*, *y*) information is collected from the sample. By adjusting the *x*, *y* position, acquisitions of the spectra from multiple points on the sample surface can be performed, assembling a spectral map of the sample (**Figure 1**). Certain techniques also have the ability to acquire the information from beneath the sample surface (*z* plane). There are essential differences among the generations of spectral imaging instrumentation; primarily, multispectral and hyperspectral techniques need to be separated. In hyperspectral approach, a large number of wavelengths are measured, yielding high spectral resolution. In contrast, in multispectral imaging only a limited number of usually very broad channels are measured whilst simultaneously recording the image. These wavelengths can be tuned for a particular application, which leads to cheaper instrumentation and simpler data processing, but without the flexibility of hyperspectral imaging systems.

**Figure 2:**
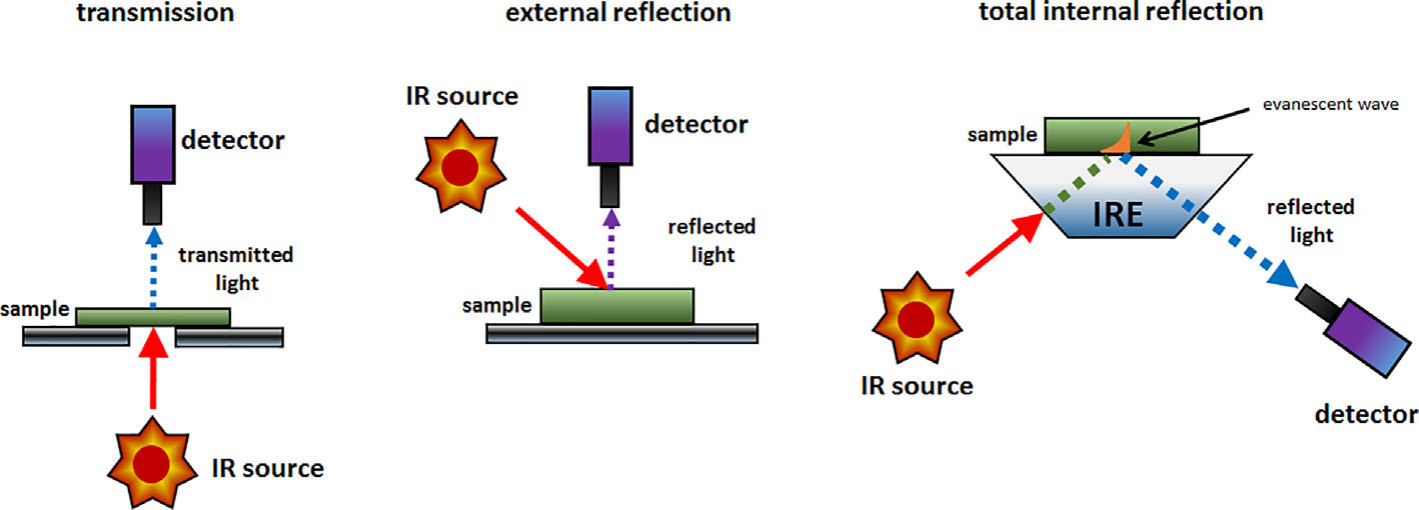
The typical sample presentation modes used in spectroscopic imaging instrumentation.

**Figure 3:**
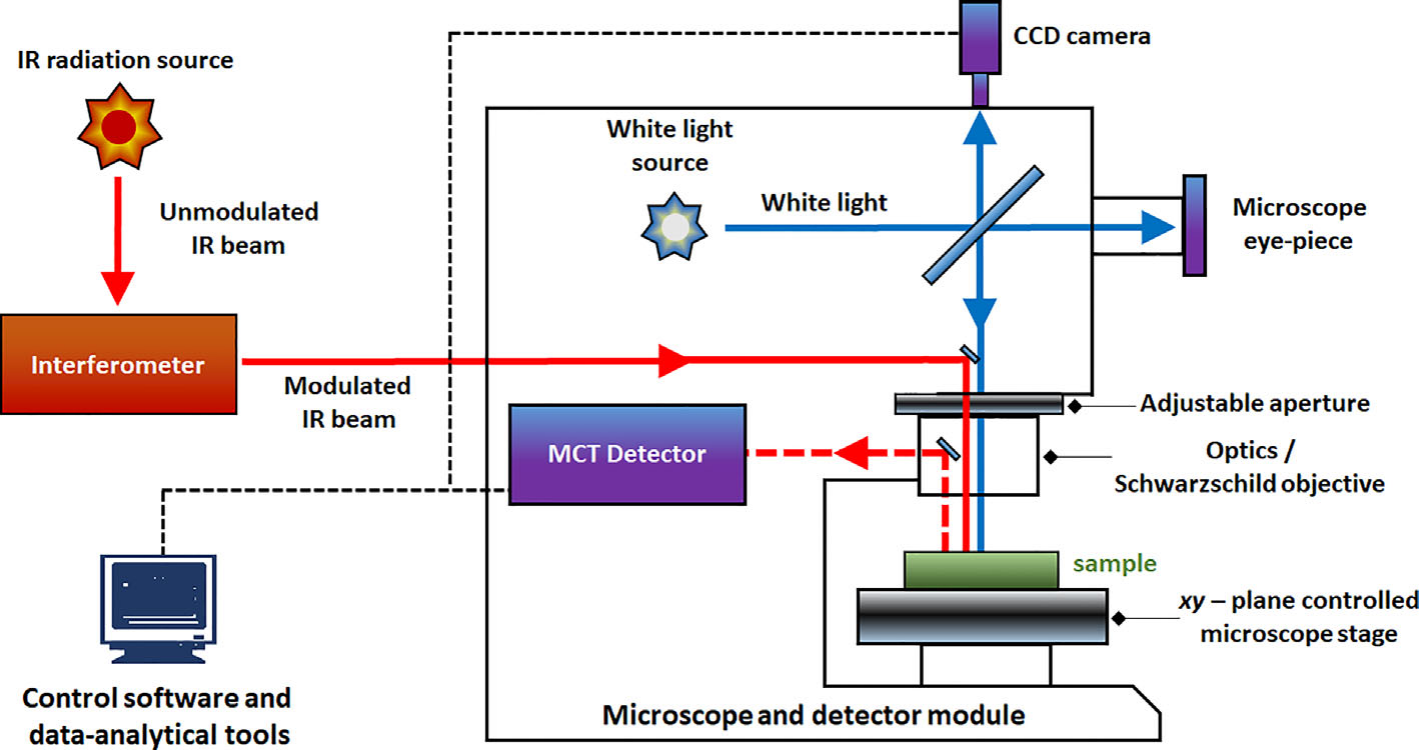
Block diagram of an FT-IR microspectrometer equipped with MCT detector and CCD camera.

The simplest implementation of a spatially resolved spectroscopy is microspectroscopy, in which a reflective optical microscope is combined with a spectrometer. For example, in an IR microspectrometer (i.e., IR microscope) the beam is focused onto a controlled point at the sample surface; this enables acquiring an IR spectrum from an extremely small sample area down to ca. 3 μm of diameter under certain conditions (Colarusso et al., 1999; Larkin, 2018). Such instrumentation uses an optical microscope system for supervision of this process. The IR spectrum may be acquired using different sample presentation modes (**Figure 2**); transmission, diffuse reflection or attenuated total reflection (ATR; i.e., total internal reflection). A chemical image of the sample may be assembled by automated process of registering point-by-point spectra from the intended area of the sample. Most commonly used are the systems utilizing diffuse reflection principle; general schematic of such instrumentation is presented in **Figure 3**. The Fourier transform (FT) instruments are preferred as their circular apertures make them better suited for integration with a microscope. Available are FT-IR microscopes utilizing conventional transmission measurements and ATR (or ‘micro-ATR’) modes as well (Larkin, 2018). Transmission mode microspectroscopic systems, although simple, face considerable limitations. Mostly, the sample thickness needs to be low enough to prevent a complete absorption of the radiation. This typically limits sample thickness to few μm, while for thicker samples only wavenumber regions featuring relatively weak bands may be acquired reliably. This often implies sample preparation, e.g., slicing, and thus excludes non-destructive way of analysis. Further complications arise if the sample surface features irregularities; its smoothness and flatness are necessary to minimize detrimental optical effects along the optical path of IR radiation (Larkin, 2018). In certain cases, this can be mitigated by encapsulating the sample between IR-transparent optical windows. In ATR approach, there is no such limitation, due the typical penetration depth of the evanescent radiation in μm range (Chan and Kazarian, 2003). However, other kinds of challenges are faced, e.g., the properties of sample-IRE contact surface strongly affect the measured spectra. To an extent, similar rules apply to measurements performed in NIR region. However, ATR approach is no longer feasible here, as the penetration depth is insufficient to yield useful spectral intensity values from weak NIR absorption. Transmission spectra can be reliably obtained from thicker samples. Moreover, in NIR reflection mode, the sample is sensed not only at its surface but deeper into its volume; this can be considered as either an advantage or disadvantage, depending on the aim of the measurement.

Further evolution of spatially-resolved spectroscopy is hyperspectral imaging (HSI). This powerful tool is based on instrumentation capable of simultaneous multi-point spectra acquisition from the sample (Kidder et al., 2006). Various scanning techniques exist in HSI, e.g., spatial scanning, spectral scanning, spatiospectral scanning, and non-scanning (Lu and Fei, 2014; Gowen et al., 2015). HSI measurement involves collection of a large amount of data in short time. Therefore, efficient architecture for data storage and processing are essential. It is commonly accepted to use the hypercube (**Figure 1**), a three-dimensional data structure that is assembled from both spatial and spectral information (*x*, *y*, *Λ*). Since hypercube is a higher-dimension structure, a reduction in dimensionality is necessary to construct a two-dimensional image; despite its critical importance for the informational value in HSI techniques, this is an immensely complex problem and only basics will be presented here. The simplest way of presenting an image can be accomplished through slicing the hypercube along a given wavelength *Λ*(e.g., the one selected as meaningful for representing the concentration of a given chemical), with each pixel coded into a false-color according to the spectral intensity, i.e., peak maximum (**Figure 1**). Other spectral features may be used in a similar way; e.g., relative intensity (spectral intensity at a given wavelength in relation to spectral intensity at another selected wavelength), integral intensity (spectrum integrated between selected wavelengths), etc. State-of-the-art image assembly is done through the methods of chemometrics, where pixel colors correspond to quantitatively resolved information on the sample property (Williams and Norris, 2001; Ozaki et al., 2006; Rinnan et al., 2009; Vidal and Amigo, 2012). Note, algorithms integrating spatial and spectral information, as well as combining information from more than one pixel are in use (Gu et al., 2008).

Briefly outlined should be the performance and quality parameters of spectral imaging, as these are crucial for practical applications. Through the introduction of the spatial dimension, additional performance parameters of an instrument need to be defined. The spatial resolution limit is related to the diffraction light limit, light scattering, and focal shifts due to high refractive index samples (Kazarian and Chan, 2010; Larkin, 2018). The diffraction-limited spatial resolution follows Rayleigh criterion, given in Eq. 1.

(1) $$r=\frac{0.61\lambda }{NA}$$

where *NA* denotes numerical aperture of the optical system and *r* is half the minimal distance at which two adjacent objects may be distinguished in the acquired image. Note, as seen in Eq. 1, the diffraction-limited spatial resolution is wavelength dependent. This implies physically-dictated difference in the spatial resolution limits between different spectral imaging modalities. Compared with the conventional point-spectroscopy, the expansion into the spatial dimension and acquisition of spectra from a very small area introduce additional challenges. Optimizations of optical throughput, source efficiency, detector sensitivity, etc, are highly important. On the example of an IR microscope, reflective optics elements yielding high-throughput transmission and focusing optical elements in Cassegrain-type configuration to mitigate optical aberrations are used. Apart of the optics, the image contrast is mostly determined by the source brightness and the type and configuration of the IR detector. Single-point detectors, linear array detectors, or two-dimensional focal plane array detectors are used. Most common IR instruments use conventional radiation sources, e.g., well-known globar (thermal light source based on silicon carbide). Noteworthy, high brightness sources are employed in new generation of instrumentation for IR imaging, e.g., tunable diode lasers, quantum cascade lasers (QCLs), or synchrotrons. High brightness sources vastly enhance spectral quality in terms of the signal-to-noise parameter (SNR or S/N; defined as the level of a desired signal to the level of background noise) and spatial resolution (Vongsvivut et al., 2019); however, such instruments are not yet widely spread and still mostly used for biomedical research. Noteworthy, macro-ATR imaging based on an inverted prism is a particularly potent technique, as it mitigates the limitations of a microscope and enables effective imaging of large sample areas (Kazarian and Chan, 2010). However, poor SNR and difficulties in aligning chemical and visual images are still an issue in ATR-IR imaging. Raman spectroscopy demonstrates advantages in these two parameters, as it combines high diffraction-limited spatial resolution with the possibility of focusing the laser on a very small spot. On the other hand, undersampling may become an issue if the spacing between acquisition points is larger than the laser spot. Unlike IR or NIR spectroscopy, Raman instruments based on FT principle are not the best option for imaging, as these require optical construction that limits the spatial resolution. Practical differences in applicability of these two techniques mostly result from the physical and chemical properties of investigated sample. A brief overview of this issue, as seen from the perspective of plant-related investigations, is presented in the *Advantages and Limitations of Spectral Imaging for Examination of Plant Tissues, Products and Related Materials*.

#### Spectra and Image Processing and Analysis

Pretreatment, processing and analysis of spectral data have key importance in generating useful image and for the understanding of the encoded chemical information. This topic is immensely complex, and only fundamentals will be briefly overviewed here. For further details, the interested reader is pointed to exhaustive literature (Norris, 1983; Massart et al., 2014; Salzer and Siesler, 2014). Spectral pretreatments are applied to suppress random variations, normalize the spectra against measurement conditions and to enhance the chemically-relevant information; these pretreatments include baseline corrections, normalizations, derivatives and smoothing (Salzer and Siesler, 2014). Image generation covers coding the spectral information into colored pixel to present it in the form useful for analysis by human. Images may be generated through simple univariate approach, in which pixels represent a single characteristic or attribute such as spectral intensity at a given wavelength; these methods require little processing power and are suitable for rapid generation of large images. However, multivariate analysis (MVA) approaches give far greater potential in elucidating chemical information as they take advantage from the excessive dimensionality of the imaging data. The most common methods include classification methods used to discriminate and group the samples depending on identified spectral variability; popular algorithms are principal component analysis (PCA), linear discrimination analysis (LDA) and support vector machine classification (SVM or SVMC). Cluster analysis methods (e.g., hierarchical cluster analysis, HCA) are often used for elucidating the spatial distribution of the spectral features (Salzer and Siesler, 2014). Plant samples constitute from multiple chemicals; in some cases, if there are only few major components, it may be attempted to decompose (deconvolute) the spectra of each of them. Unmixing approaches such as multivariate curve resolution (MCR methods), e.g., MCR alternative least squares (MCR-ALS) should be mentioned here. Quantitative correlation of the spectral data with reference quantities (e.g., concentration of a given chemical) may be performed with multivariate regression (Burger and Geladi, 2006). Partial least squares (PLSR), multiple linear (MLR), and principal component (PCR) regression algorithms are noteworthy. Once an image is generated, additional processing is available. For instance, cross-pixel information can be used for further gains. Discussions of the relevant topics covering image processing, reduction of data dimensionality and image fusion are available in literature (Burger and Gowen, 2011; ElMasry and Nakauchi, 2016; de Juan et al., 2019).

### The Advantages and Limitations of Spectral Imaging for Examination of Plant Tissues, Products and Related Materials

Spectral imaging is an extremely potent tool in plant-related field of research, given its capability for performing *in-situ* non-intrusive compositional and functional analysis in the form of a surface and sub-surface map of the sample (Türker-Kaya and Huck, 2017). Additionally, point-specific quantitative information on the sample is available, e.g., the content and distribution of a certain molecule of interest can be obtained. Given the structural and chemical complexity of plant organs, these capabilities add up to form an outstanding exploratory potential common for all of the techniques reviewed here. Nevertheless, different approaches to imaging that result from the differences in the physicochemical foundations of the spectral techniques, design and engineering principles of the instrumentation, or sampling methods among other factors, separate the applicability of spectral imaging modalities in various plant-related kinds of research. Depending on the sample type, measurement conditions and aims of the analysis, different spectral imaging techniques may be preferable. **Table 3** summarizes the primary parameter ranges of the reviewed techniques, typical values of the working spectral region, spectral and spatial resolution.

**Table 3:** Typical working parameters of hyperspectral imaging instrumentation relevant to the reviewed topic.

Spectral technique	Spectral parameters			Spatial resolution
	Spectral region(wavelength)	Spectral region(wavenumbers)	Resolution	
Vis/NIR	400–1,000 nm	25,000–10,000 cm^-1^	1.5–5 nm	0.25 – 1.336 μm
IR	3,280–12,500 nm	3,050–800 cm^-1^	4 cm^-1^	3.2–10 μm
Raman	2,855–33,333 nm	3,500–300 cm^-1^	2.6–4 cm^-1^	0.25–1 μm
Fluorescence	500–800 nm	12,500–20,000 cm^-1^	3–5.6 nm	0.2–0.45 μm

IR imaging offers high chemical specificity and relative ease of tracing chemical constituents in the sample. It has reasonably high sensitivity and the instrumentation in its basic form (i.e., IR microscope) is relatively simple. However, IR spectra are easily obscured by the presence of water in the sample, and hence, IR external reflection or transmission techniques are limited in studies *in situ* and in examinations of not dried plant material. Additionally, IR measurements are sensitive to the presence of atmospheric gases [ro-vibrational structure of H_2_O(g) and CO_2_(g)], which may become problematic in the setups where the radiation beam travels through open air. Therefore, external measurement conditions need to be controlled with greater care than in other techniques. Moreover, IR imaging instrumentation is rather restricted in spatial resolution because it operates over relatively long wavelengths (**Table 3**). Further, near the limit of the optical resolution the effective optical throughput drops significantly and the SNR parameter decreases. Compared with IR, the loosened diffraction limit resulting from a shorter wavelength of NIR radiation yields a relatively higher contrast of spectral images. Shorter NIR wavelengths enable imaging instrumentation to achieve better spatial resolution. In addition, high spectral resolution is not as much stressed, as NIR bands are broad and loss of chemical information in low-resolution is manageable. This, combined with the availability of rapid scanning detectors, makes NIR imaging instrumentation simpler. NIR and IR imaging techniques are similar to an extent and even the instrumentation capable of working in both regions is available on the market. The key practical differences in applicability of these two techniques root in their physical background. Because of extensive band overlapping, NIR spectra tend to be much more difficult for direct interpretation and chemical specificity of NIR images may vary, depending on the chemical composition of the sample, with enhanced signature of some components (X-H chemical groups). Water has very strong absorption bands in IR region, which overlaps with a number of characteristic bands of organic compounds (**Table 1**). Therefore, IR imaging is not optimal for examining moist samples, while NIR is relatively less affected. Distinct difference between the typical NIR and IR absorption coefficients results in rather deep probing of the sample by NIR radiation (several millimeters beneath surface), while IR tends to probe the surface (penetration depth in μm range).

The characteristics of Raman spectroscopy lead to certain advantages in imaging application. Laser light can easily be focused on a small spot yielding high spatial resolution, e.g., in confocal Raman microscope. Therefore, very small sample volumes can be studied (< 1 µm in diameter, < 10 µm in depth). Theoretical resolution level resulting from diffraction limit are favorable, e.g., a 532 nm laser with a 0.90/100x objective corresponds to a spatial resolution of 0.36 μm; in practice, due to complex optical effects the resolution is ca. 0.5 μm. Confocal instruments offer unique depth resolution with the possibility to probe the sample beneath its surface. In such case, image acquisition at controlled depth dimension *z* is available; hence, the collected hyperspectral data has (*x*, *y*, *z*, **λ structure. However, interrogation of plant sample meets additional limitation here, as sharp difference between the refractive indices of the cell walls and cytoplasms, and abundance of pigments and fluorescent molecules disrupts the transmission of the light in the sample. Effectively, under typical conditions the penetration depth of light in plant tissue is limited to ca. 30 μm, which corresponds to the distance of only a few layers of cells. Moreover, the listed phenomena can be detrimental for image quality (Feijó and Moreno, 2004; Paddock and Eliceiri, 2014). For dried samples the danger of thermal decomposition due to energy delivered by the excitation laser at a sample spot should be mentioned. Balance needs to be found between a suffcient intenisty of the Raman signal and heat damage induced in the sample; this factor can be controlled by optimizing the exposure time, laser power, shape and size of the laser spot at the sample surface (Vítek et al., 2020). Moist samples are easily studied, as the spectral information is not obscured by water signal and living plants can be investigated. However, a considerable disadvantage results from stimulated autofluorescence emission from chlorophyll, as its signal tends to obscure all other molecules in ‘green’ plant samples. One of few exceptions are carotenoids, abundant pigments in numerous plants, for which resonance condition can conveniently be achieved, e.g., their *v*_1_(C=C) band becomes strongly enhanced in Raman spectrum. By performing two independent measurements simultaneously using two excitation lasers with different wavelengths, fluorescence and Raman spectra can be discriminated; however, this approach requires more complex instrumentation (Cooper et al., 2013). Wide-field illumination Raman is a multi-spectral imaging approach, in which only selected Raman shifts (wavelengths) are measured; therefore, the collected chemical information is scarcer. Such instrumentation is simpler, and relatively fragile specimen may be examined as the energy of excitation laser that reaches the sample is dispersed over the scanned sample area (wide field-of-view). The problem of thermal damage induced to sample by the excitation laser has prime importance for studies of delicate plant specimens. Even relatively mild heating at the laser spot occurring in Raman confocal microscope can affect the structure of biomolecules, in particular proteins. On the other hand, the exposition parameters are highly important for yielding quality images. The optimization of the measurement conditions is a continuously developed topic. Recently, Hauswald et al. (2019) dissected the existing strategies for sample illumination and proposed a novel approach that improves Raman imaging quality versus the thermal illumination limit. The study evaluated the practical applicability of point- and line-confocal microscopes as well as widefield-, light sheet-, and light line illumination, based on the developed models describing the fundamental physical limits of Raman spectroscopy with respect to SNR, sample load and maximum imaging speed. These accomplishments may help to develop new concepts of Raman microscopy, by extending its applicability for the three-dimensional measurement of biological samples including large and sensitive specimens (Hauswald et al., 2019).

Fluorescence imaging occupies a particular spot across the field of plant-oriented studies. On the one hand, the abundance of chlorophyll as a strong fluorophore obscures most of the signal of other chemical constituents in the imaging of plant-related samples. Fluorescence spectra are less specific than IR, Raman, or even NIR spectra. Thus, in this case the ability to obtain chemical profile of the sample is largely inferior to that of the previously discussed techniques. Chemical clearing from chlorophyll and pigments, and subsequent staining with fluorescent die, enables imaging of internal structures through highlighting cell walls. On the other hand, that feature of chlorophyll makes fluorescence imaging applicable to living plants as a sensitive tool capable of monitoring plant’s metabolism (chlorophyll fluorescence imaging, CFI). It is a highly accurate indicator of the photosynthetic efficiency, which makes fluorescence imaging a very potent tool in plant science research (Warner et al., 2014), as it unrivaled unique insights into plant phenotypic variation (Meijón et al., 2014), gene expression patterns (Truernit et al., 2008), or plant-microbe interactions (Dagdas et al., 2012). Since the instrumental principle is similar to that used in Raman imaging, with laser excitation, this technique features similar optical advantages. Moreover, three-dimensional fluorescence imaging is available. Proteins are sensitive fluorophores as well, and their subcellular distribution alongside nonproteinaceous cellular constituents can be visualized with this technique.

To mitigate the limitations in light penetration distance through micro-structured, heterogeneous medium such as a plant sample, mechanical sectioning or clearing with chemical agents is routinely applied. These conventional approaches enable imaging of internal plant structures. However, they remove the non-intrusive character of analysis, need to be performed carefully to avoid introducing artificial damages and changes in the sample and, sectioning in particular, are labor and time intensive. Alternative clearing methods with fewer drawbacks have been developed and are discussed in *Fluorescence Imaging*.

## Applications of Spectroscopic Imaging in Plant-Oriented Studies

### IR Imaging

#### Investigations of Microstructural Features

**Figure 4:**
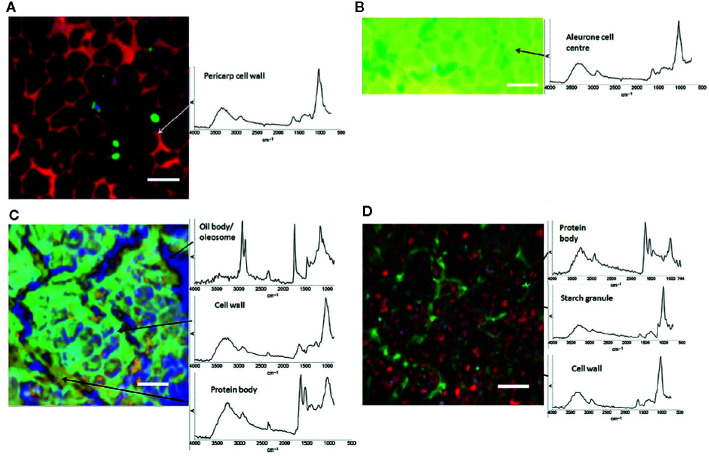
Images of hand-dissected wheat kernel tissues (Consort), shown as false-color principal component analysis (PCA) images, with example spectra provided for regions of interest: **(A)** pericarp-testa; **(B)** aleurone; **(C)** germ; and **(D)** endosperm. Scale bars: 50 μm. Reproduced in compliance with CC BY 4.0 license from Warren et al., 2015.

IR microspectroscopy is a well-established powerful tool used for investigations of the microstructure, chemical composition and functionality of plants at a subcellular level. Despite a relatively unfavorable level of diffraction limit, it remains within the reach of high-performing IR instrumentation to resolve cellular and sub-cellular structures in plant samples. On the other hand, relatively high chemical specificity of IR spectra gives such studies the necessary fingerprinting capability. For example, Warren et al. (2015) demonstrated their successful application of IR imaging system in resolving individual cells and cell walls in the images acquired from common wheat (*Triticum aestivum*) kernels and *Arabidopsis* sp. leaves. Furthermore, large structures within cells, such as starch granules and protein bodies, were clearly identified. This required sufficient contrast, which was achieved through PCA overlays and correlation analysis applied to hyperspectral data cubes to generate images. Unsupervised PCA algorithm was sufficient to generate a clear image of the sample microstructure (example provided in **Figure 4**), while the correlation analysis enabled confirming the identity of different anatomical structures based on the spectra of isolated components. The proposed approach allowed distinguishing gelatinized and native starch within the cells. Further, the loss of starch during wheat digestion, as well as the accumulation of starch in leaves during a diurnal period could be clearly evidenced in the generated images (Warren et al., 2015). Correspondences between chemical, microstructural and mechanical properties of cell walls were investigated by IR imaging as well, e.g., the properties related to microplasticity were studied by Largo-Gosens et al. (2014).

Cell walls are highly characteristic microstructural features of plant tissues. Noteworthy, cell walls attracted much attention in IR imaging studies relatively early (McCann et al., 1992; Suutarinen et al., 1998). This technique demonstrated particular potential for elucidating structural and functional properties of cell walls. IR spectra in the region of 1200-950 cm^-1^ contain several characteristic peaks of the cell wall constituents (Schulz et al., 2014). For example, Monti et al. (2013) reported that IR absorption at the following wavenumbers can be correlated with the molecular components of cell walls; 1,740, 1,595, 1,440, 1,150, 1,105, and 975 cm^-1^ (pectins); 1260, 1230 and 1075 cm^-1^ (hemicellulose); 1025 cm^-1^ (cellulose); additionally, refer to **Table 1** for group frequencies. This advantageous characteristic of IR spectral techniques can be used with high success to investigate the chemical structure of cell walls (Carpita et al., 2001; Barron et al., 2005; Dokken and Davis, 2007; Gou et al., 2008; Saulnier et al., 2009; Gorzsas et al., 2011; Zhong et al., 2011; Monti et al., 2013; Pesquet et al., 2013). Furthermore, through patterned shifts and intensity variations the absorption bands can deliver information on the chemical environment and intermolecular interactions of the molecules within the cell walls. This feature enables monitoring the alterations in cell wall components in different tissues in connection with physiological changes or throughout plant development and growth. For instance, endosperm textures of hard and soft wheat (*Triticum aestivum*) were studied by Barron et al. (2005). The presence of higher amounts of a water-extractable arabinoxylan in the peripheral endosperm of soft grains was unveiled. Cell elongation, e.g., during growth, affects the molecular structure of the wall. This phenomenon as investigated, e.g., by Carpita et al. (2001) in maize (*Zea mays*), or in grains as reported by Saulnier et al. (2009). During embryo generation, it was evidenced that chemical composition of cell walls varies; an increase in cellulose (identified at 900 and 1,320 cm^-1^) and a decrease in pectin (identified at 1,014, 1,094, 1,152, 1,238, and 1,741 cm^-1^) was observed by Zhong et al. (2011). These features may be useful markers for imaging studies (Gou et al., 2008). Biochemical changes in cell walls occurring in leaves were observed by Gou et al. (2008) and concluded to correlate with the leaf maturity. Authors observed acetyl esterification of the cell walls in black cotton-wood (*Populus trichocarpa*). For young leaves accumulation of *p*-coumarate was characteristic, while its content decreased in mature leaves. Post mortem, changes could also be monitored by IR microspectroscopy, e.g., lignification of treachery elements of common zinnia (*Zinnia elegans*), in which case the characteristic wavenumbers 1510 and 1595 cm^-1^ were established as the markers of the chemical changes (Pesquet et al., 2013). Recently, novel insights into cell wall chemistry at cellular level have been obtained by Cuello et al. (2020) using ATR-IR microspectroscopy. They succeeded in non-destructive microphenotyping of the three types of popular wood; normal wood of staked trees, tension and opposite wood of artificially tilted trees. Cell wall composition could be dissected with respect to the cell wall multi-layered structure, gelatinous extra-layer (G-layer), S2 and S3 layers (S-layers). These continuously developing studies evidence the potential or IR imaging to monitor the temporal and spatial patterns in biochemistry, physiology, and microstructural features of plants during their development. IR imaging has become fairly matured tool in investigations of cell walls (Schulz et al., 2014), with research activity being shifted towards applications of Raman imaging technique (Prats Mateu et al., 2020). Nonetheless, recent attention is given to increasingly accessible IR imaging instrumentation utilizing high brightness radiation sources. The limitation in spatial resolution of the conventional IR microspectroscopy can be significantly lifted by employing synchrotron radiation (SR) source. The improved spatial resolution and SNR compared with conventional IR imaging substantially enhances the potential for examination of microstructures in plant samples, unveiling subcellular details unachievable with traditional approach. In this field of application, SR-IR imaging shares some of the advantages of the techniques based on Raman spectroscopy. As reported by Butler et al., 2017, SR-IR imaging performs notably better in the analysis of living plant tissues, as the detrimental effect introduced by the presence of water in the sample can be minimized using this technique. It eliminates the need for time-consuming sample preparation (i.e., tissue fixing) and directly improves the research potential by enabling studies of plant tissues in native state. This potential was utilized by Butler et al. (2017) as demonstrated by their ability to detect calcium (Ca) deficiencies in *C. communis* leaf samples.

#### Spatial Distribution of Chemical Composition in Plants

**Figure 5:**
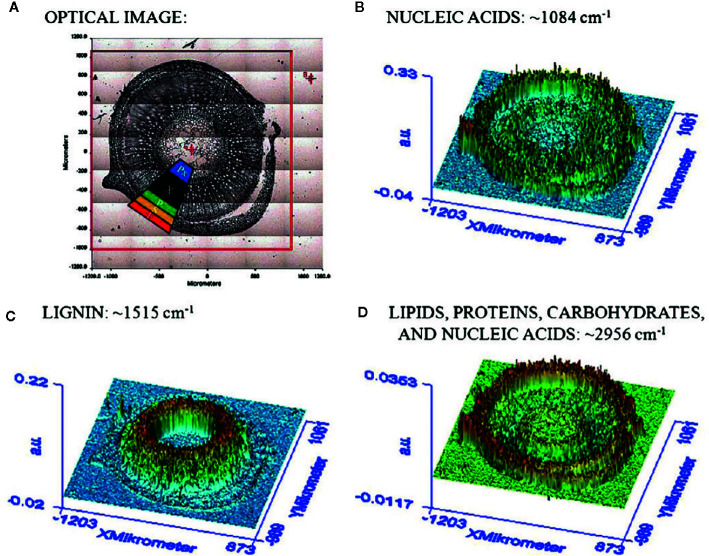
**(A)** Section through the caulis of St. John’s wort (*H. perforatum*) with marked regions (2) obtained by Huck-Pezzei et al., 2012. (e) epidermis, (s) sclerenchyma, (p) phloem, (Px) protoxylem, and (x) xylem. **(B)** FTIR imaging result shown in false color representation. Colors reflect intensities of the selected absorption at 1084 cm^−1^, which is commonly attributed to nucleic acids. **(C)** FTIR imaging result shown in false color representation. Colors reflect intensities of the selected absorption at 1,515 cm^−1^, which is commonly attributed to lignin. **(D)** FTIR imaging result in false color representation. Colors reflect intensities of the selected absorption at 2,956 cm^−1^, which is commonly attributed to lipids, proteins, carbohydrates, and nucleic acids. Reproduced with permission (Springer) from Huck-Pezzei et al., 2012.

**Figure 6:**
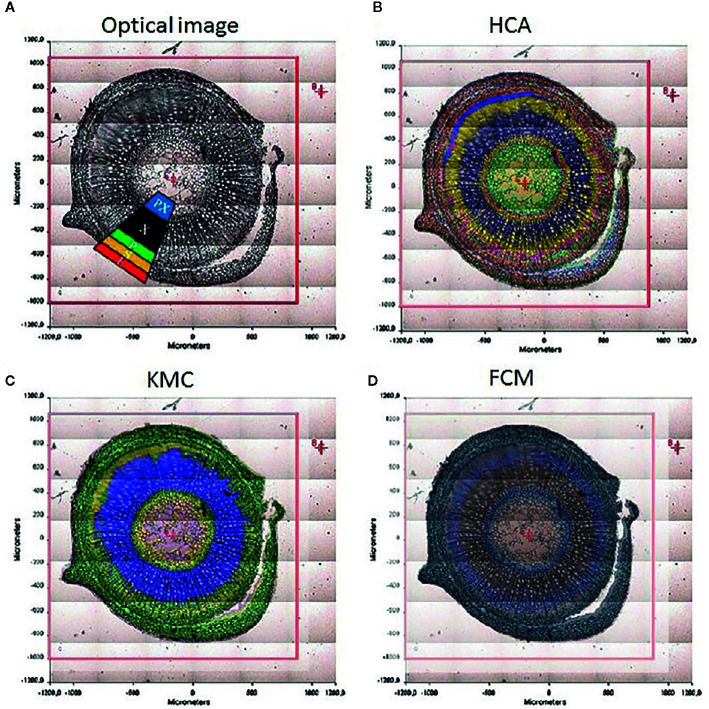
The significance of spectral image processing method for elucidating the chemical information from plant specimen. **(A)** section through the caulis of St. John’s wort (*H. perforatum*) with marked regions; **(B)** hierarchical cluster analysis; **(C)** k-means clustering image; **(D)** spectroscopic image of the fuzzy c-means clustering. Reproduced with permission (Springer) from Huck-Pezzei et al., 2012.

The chemical specificity of IR spectroscopy makes the corresponding imaging techniques applicable directly to study the spatial distribution of biomolecules in plant tissues. The most essential gain here is the determination of distributions of chemical compounds in plant tissues quantitatively. IR imaging was demonstrated relatively early to be a potent tool in this role. For instance, Huck-Pezzei et al. (2012) could successfully identify the characteristic wavenumbers and use them to monitor the distribution of several classes of major biochemical constituents in tissues of St. John’s wort (*Hypericum perforatum*); lipids (1,740 cm^-1^), phospholipids (1,240 cm^-1^), proteins (1,630 and 1,550 cm^-1^), carbohydrates (1,185 to 930 cm^-1^), and nucleic acids (1,080 cm^-1^). Furthermore, distribution of these molecules was successfully determined in epidermis, phloem, protoxylem, sclerenchyma, and xylem tissue (**Figure 5**). As evidenced by comparative analyses of their data, in the study by Huck-Pezzei et al. (2012) the spectral data processing and interpretation was essential to resolve reliable and useful information on the spatial distribution of chemical information (**Figure 6**). In sharp contrast to an optical image or univariate spectral analysis, clustering techniques including hierarchical cluster analysis (HCA), k-means clustering (KMC), and fuzzy C-means clustering (FCM) are capable to significantly improve one’s ability to interpret IR imaging data collected from plant specimens. In the discussed case, clustering techniques algorithms increased the information content of the IR images dramatically and enabled differentiating morphological and molecular patterns of different tissues. Huck-Pezzei et al. (2012) could semi-quantitatively determine the distribution of chemical ingredients such as lipids, phospholipids, proteins, carbohydrates, nucleic acids, and cellulose in the images of different tissue types.

Similar chemical profiling and characterization of the spatial distribution of several constituents present in *Ginko bilboa* leaves was performed by Chen et al. (2013). Interestingly, that study involved ATR-IR and NIR imaging techniques and demonstrated well the practical difference between both approaches in the analysis of plant tissue. The superior potential of the former one to provide chemical fingerprinting of the sample was noted. Through the analysis of the characteristic IR bands, distribution of, e.g., proteins, saccharides, esters, glycosides, ketones, oxalates, aromatic, unsaturated and long chain aliphatic compounds could be performed. However, the limitation of the elucidated information to the sample surface in ATR-IR technique made use of the deeper sampling characteristic of NIR imaging highly useful in that case. The latter technique enabled rapid exploration of the distribution of the primary chemical constituents in a whole leaf blade. Authors concluded that the combination of both imaging techniques yield the highest exploratory potential. It was also stressed, that ATR-IR approach does not require sample preparation (i.e., microtoming) necessary for IR transmission measurements, which is an essential advantage in plant tissue analysis, as no chemical reagents are used that can change the native composition of the tissue, nor cutting that can mechanically distort the structure of the tissue or cause migration of chemical constituents.

The concept of combined use of ATR-IR and NIR imaging spectroscopy was continued by Chen et al. (2015). That study focused on enhancing the spectra analysis methods towards improved fingerprinting capability. In addition to several MVA methods (PCA and independent component analysis-alternating least squares, ICA-ALS; partial least squares target, PLST), two-dimensional correlation spectroscopy (2D-COS) was applied as well. Detailed distribution maps of eugenol and calcium oxalate in tissue sample of calyx tubes (from dried bud) of clove (*Eugenia caryophyllata*) demonstrated the potential of elucidating chemical information from spectral images featuring substantial band overlapping. Noteworthy, it was shown by Chen et al. (2015) that simultaneous application and analysis of both techniques improves interpretability of NIR spectral images. Similar potential was demonstrated in exploring the chemical morphology of areca (*Areca catechu*) nut (Chen et al., 2016).

#### Investigation In Vivo of the Properties of Biomolecules

Further, high sensitivity and selectivity of IR spectroscopy towards different chemical compounds enables determination the physicochemical properties of biomolecules *in vivo*. Determination of protein structure is well established, as amide I and amide II bands (**Table 1**) are particularly characteristic markers of that feature (Kumar et al., 2016). Protein structure is sensitive to local environment and can be used to sense the physiological state of the organism. On the other hand, protein structure correlates to nutrition quality of certain crops, and is meaningful for livestock digestive behavior and nutrient availability, which adds up to the topic’s significance in agriculture-related research. Relevant examples include the exploration of barley protein by Yu (2006), wheat protein by Bonwell et al. (2008); attention should be given to the applications of synchrotron-based IR imaging technique (e.g., by Yu et al., 2009 and Xin et al., 2013). Highly-sensitive synchrotron radiation-based Fourier transform infrared microspectroscopy (SRFTIRM) was applied by Yu et al. (2009) to investigate protein structures in agricultural plant forage. The distribution of those structures influences nutritional value of protein, as e.g., beta-sheet proteins have limited access to gastrointestinal digestive enzymes. HCA and PCA analyses identified protein alpha-helices, beta-sheets and other structures such as beta-turns and random coils in SRFTIRM imaging data collected from maize specimens (Yu et al., 2009). Synchrotron IR imaging system operating with high spatial resolution (10x10 μm) was also employed by Xin et al. (2013) to examine *in vivo* protein chemical characteristics and secondary structure and carbohydrate internal structure, with respect to chemical differences in wheat specimens. Normal and frost damaged wheat was examined, with aim to dissect structural variation and frost-induced damage, as these factors have critical importance for the nutritional value of wheat. IR fingerprints of protein and carbohydrates could be identified in the generated images; protein amide I and II bands (ca. 1,774–1,475 cm^-1^), structural carbohydrates (SCHO, ca. 1,498–1,176 cm^-1^), cellulosic compounds (CELC, ca. 1,295–1,176 cm^-1^), total carbohydrates (CHO, ca. 1,191–906 cm^-1^), and non-structural carbohydrates (NSCHO, ca. 954–809 cm^-1^). Evidences of frost-induced damage were gathered based on the spectral variations among the studied wheat grain specimens. Frost damaged wheat revealed suppressed amide I and II bands, as well as the bands due to carbohydrate-related functional groups, including SCHO, CHO, and NSCHO. The intensity ratio of protein bands and some of carbohydrate bands was also observed. The study suggested that chemical changes and structural variations in wheat and other grains influenced by climate conditions, such as frost damage, might be a major reason for the decreases in nutritive values, nutrients availability and milling and baking quality in wheat grains. These conclusions are significant for exploring the effects of cultivation conditions and external factors on the protein matrix of agricultural plants, and their resulting nutritional values.

Characteristic IR wavenumbers were successfully used by Gonzalez-Torres et al. (2017) to identify proteins (amide I peak at 1630 cm^-1^) and carbohydrates (C-O peak at 1030 cm^-1^) distribution in algae single cells and algal organic matter (AOM) extracts of *M. aeruginosa* and *C. vulgaris*. Elucidation of the composition and distribution of these biomolecules gave insight into the chemical interactions that drive physical floc properties. *C. vulgaris* formed larger flocs characterized by homogenous distribution of proteins and polysaccharides across its area, while smaller flocs of *M. aeruginosa* were observed to develop localized areas of increased protein concentration. The latter features tended to be present near the edge of regions absent of biomolecules, where the coagulant was expected to appear. The interactions between the investigated biomolecules were considered. High Pearson’s correlation between carbohydrates and amides was determined in the imaging data of *M. aeruginosa*. Either the presence in the cell surfaces of peptidoglycans characterized by amide groups linked to carbohydrates or the interaction between peptides and carbohydrates at the level of macromolecular pool were suggested as the feasible origins of that observation. In light of the earlier literature, Gonzalez-Torres et al. (2017) hypothesized that proteins not conjugated with carbohydrates form complexes with coagulant in *M. aeruginosa* flocs. Such binding was not observed in the case of *C. vulgaris*, and this was proposed as the main driver in forming stronger, more uniform floc by this species. IR imaging study yielded valuable insight on how the inter actions between biomolecules and the distribution of biopolymer, proteins, and carbohydrates influences cell micro-aggregation in different algae species.

#### Biochemistry of Adaptive/Defensive Mechanisms in Plants

IR imaging is useful in monitoring biochemical changes underlying the adaptive and defensive mechanisms employed by plants in response to external perturbations, e.g., pathogens. Synchrotron IR imaging combined with atomic force microscope infrared spectroscopy (AFM-IR) was recently employed for investigation of the formation of extractive-rich heartwood (HW) in live trees at cellular level (Piqueras et al., 2020). The role of this natural mechanism is to increase tree’s resilience against fungal degradation; however, little was known before about the deposition pathways and the distribution of extractives. In the discussed study, imaging data was acquired from Kurile larch species (*Larix gmelinii* var. *Japonica*) across the HW formation zone sampled through transverse and tangential micro-sections of wood. MCR-ALS deconvolution algorithm was used for unmixing purposes. IR spectral signatures were successfully resolved, with major spectral changes occurring in the transition zone from sapwood to HW. A decrease in the absorbance at ca. 1,660 cm^-1^ and an increase of the absorbance at ca. 1,640 cm^-1^ were identified. Several possibilities were suggested for interpreting this pattern, with type II (Juglans-type) process suggested as the most viable, where an underlying accumulation of phenolic precursors in the sapwood rays precedes extractives oxidation and condensation in the transition zone from which they spread to the neighboring HW cells (Piqueras et al., 2020). Noteworthy, IR imaging provided valuable insight into the importance of the local environment on the response mechanisms at single-cell level (Op De Beeck et al., 2020). While that study concerned fungi (Basidiomycete *Paxillus involutus*), it may be expected that plant cells feature a similar behavior dependent on the microenvironment. This was concluded based on the fungi decomposition activity that was observed to be regulated by the local conditions.

### NIR Imaging

#### Water Distribution in Plant Body and in Soil

Water is an essential substance for functioning of any plant, and the knowledge on its distribution in proximity of a plant brings key benefits for several disciplines of science. It is meaningful for basic understanding of plant physiology, but also substantially aids our ability to counter drought in practical agricultural applications by improving crop water uptake capacity and maximizing the yield. As explained in *The Advantages and Limitations of Spectral Imaging for Examination of Plant Tissues, Products and Related Materials*, the characteristics of NIR spectroscopy make it particularly suited for analysis of the presence of water in sample. In this role, it is far superior to the popular multispectral Vis (RGB) imaging, which is not sensitive towards water signal. On the other hand, NIR light penetrates deeper and is not prone to complete absorption, unlike in IR techniques. These advantages of NIR-HSI approach could have been successfully employed by Arnold et al. (2016) for examination of the distribution of water in roots of living plants and soil. Compared with approaches based on Vis region, the study confirmed a supreme level of chemical information unraveled in an NIR image of drought-resistance roots. Substantially improved image contrast that directly enabled segmenting roots from soil, discrimination of the essential plant features by their unique spectral characteristics, acquisition of additional information, e.g., on root structure, were concluded as the gains offered by NIR-HSI experiment. The capability to study the water distribution within the body of a plant offers key benefits for plant science, and it may be expected that feasible methodologies towards such goal will continue to develop.

Recent research activity at this direction demonstrated the potential of NIR-HSI to perform spatially-resolved quantitative analysis and visualization of moisture content distribution in tea leaves (Sun J. et al., 2019). A sophisticated suit of data-analytical methods was employed for this purpose, successive projections algorithm (SPA) coupled with stepwise regression (SPA-SR), and competitive adaptive reweighted sampling (CARS) coupled with stepwise regression (CARS-SR). Further, the study involved several spectral image preprocessing methods coupled with feature selection algorithms. Twenty combined treatments were evaluated to yield the best prediction models based on multiple linear regression (MLR). The highest performance of MLR model was obtained when the spectral images were pretreated using Savitzky-Golay and multiplicative scatter correction (SG-MSC) combined with CARS-SR. This approach retrieved best the distribution of moisture content in tea leaves. The established suit of methods for obtaining water distribution maps in the leaf tissue offered new insights essential for improving plant irrigation methods.

While NIR spectroscopy is intrinsically better suited than IR for examining water distribution in biological tissue, it should be mentioned, imaging technique based on terahertz (THz) spectroscopy is a capable tool for this purpose as well (Gente et al., 2013; Gente et al., 2018). Recently, THz time-domain imaging provided three-dimensional mapping of water distribution in a leaf tissue of succulent plant *Agave victoriae-reginae* (Singh et al., 2020).

#### Analysis *In Vivo* of Macro- and Micronutrients

In plant science, the vast majority of spectral imaging studies were aimed for high throughput plant phenotyping, in which the pursued information corresponded mostly to specimen morphological, such as size or growth, and physiological features (for instance, chlorophyll Photosystem II). HSI technique is capable, however, to determine the spatial distribution of chemical contents within plant specimen as well. For example, Yu et al. (2014) developed a method for determining the spatial distribution of total nitrogen content (TNC) in pepper plant based on Vis/NIR imaging. As a non-intrusive analysis, it has a critical advantage over the conventional destructive methods, such as Dumas combustion method, as it enables *in vivo* studies. The above discussed example concerned an analysis of a single chemical property in a plant. However, it has been demonstrated by Pandey et al. (2017), that through employing HSI system operating over a broad spectral region covering Vis and NIR (18,182–5,882 cm^-1^; 550–1,700 nm) it is feasible to acquire *in vivo* quantitative information on various chemical properties. The discussed study examined maize and soybean plants, each in 60 samples, towards water content and macronutrient concentrations. The latter included nitrogen (N), phosphorus (P), potassium (K), magnesium (Mg), calcium (Ca), and sulphur (S), and micronutrients sodium (Na), iron (Fe), manganese (Mn), boron (B), copper (Cu), and zinc (Zn). The specimens were exposed to biotic stress, either through water deficit or through nutrient limitation, to introduce variable properties in the sample set. With exception of Na and B, all other concentrations could have been successfully quantified by PLS models, although the prediction performance for micronutrients was comparatively lower, which seems reasonable giving their lower concentration profiles. This accomplishment demonstrated the potential of Vis/NIR imaging to perform high-throughput nutrient quantification in living plants.

A considerable attention was given to the analysis of nitrate in plant tissue. Nitrates are essential for plant physiology; however, excessive fertilization may lead to an unhealthy high nitrate content in vegetables. Yang et al. (2017) applied NIR-HSI technique to gain better understanding of the spatial distribution of nitrates in spinach leaves. Images were collected *in vivo* and subjected to PLS regression analysis to yield quantitative per-pixel information on nitrate content. The approach was demonstrated to be capable of providing high-resolution maps of nitrate content in different parts of spinach leaves. Detailed display of the nitrate distribution in the petiole, vein, and blade was possible. Noteworthy, the study revealed dynamic changes in the nitrate content in intact leaf samples under different storage conditions. Therefore, the method shows value as a rapid, high-resolution non-destructive tool for quantitative analysis of the nitrate content, and distribution in vegetables.

Development of quantitative analytical methods based on NIR-HSI for elucidating nutrient distribution and dynamics in plants remains an active field of research. Wang et al. (2020) used HSI instrumentation operating in 908–1735 nm wavelength region (11,013–5,763 cm^-1^) to monitor P and K contents in tea plant leaves. The study involved 87 leaf samples from five different cultivars. A considerable attention was given to the selection of the best performing spectral image pretreatment methods, with prime aim to eliminate the detrimental effect of noise in raw spectral data. Subsequently, the accuracy of the prediction of P and K contents through several MVA prediction methods were compared. The best results were obtained with the use of standard normal variate (SNV) for spectra pretreatment and successive projections algorithm coupled with multiple linear regression (SPA-MLR) to predict the contents of P and K content in tea leaves.

#### Quality Assessment of Plant Products

**Figure 7:**
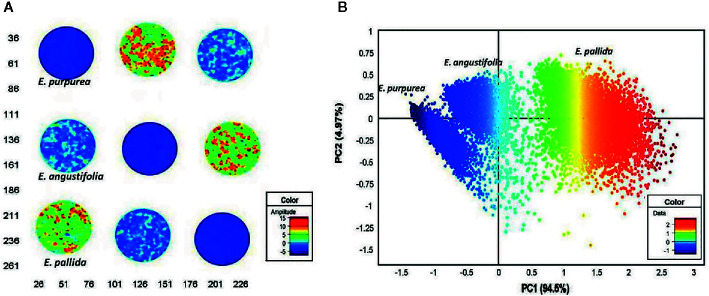
Principal component analysis (PCA) score image (t1) of *Echinacea* sp. leaf powders based on color amplitudes **(A)**. The corresponding score plot (PC1 vs. PC3) shows minimal separation of the pixel clusters **(B)**. (EAL—*E. angustifolia* leaf, EPL—*E. purpurea leaf*, EPaL—*E. pallida* leaf). Reproduced in compliance with CC BY-NC-SA 3.0 license from Sandasi et al., 2014.

Quality control plays a critical role for medicines derived from plants, as the chemical composition of natural drugs is prone to a much greater variation than conventional pharmaceutical products. In this field, spectral imaging offers substantial improvements over the classical methods. This potential has been demonstrated, e.g., by Sandasi et al. (2014) by their method developed for authentication of *Echinacea* based medicines appearing on the pharmaceutical market. *Echinacea* species are often included in various formulations to treat upper respiratory tract infections. The study involved three species, *E. angustifolia, E. pallida*, and *E. purpurea*, acquired from local market in South Africa. By employing NIR-HSI technique operating in 1,0870–3,978 cm^-1^ (920–2,514 nm) region, with aid of PCA it was possible to clearly discriminate between the three *Echinacea* species and further differentiated the roots of different specimens (**Figure 7**). The method accurately predicted the raw material content in several commercially available products and identified products that did not contain crude *Echinacea* material as well.

Herbal teas often constitute of blended plant species and it is critical to be able to determine these constituents in order to assess the tea quality. In sharp contrast to destructive, manpower- and time-intensive conventional routes of analysis (e.g., chromatography), NIR-HSI offers rapid and non-destructive alternative, with addition of rich chemical information and its spatial distribution. It was demonstrated in literature, that NIR-HSI is a potent technique for controlling quality of herbal teas, e.g., as reported by Djokam et al. (2017) on their successful discrimination between tea blends of different brands with varying quality and origin. The quality parameters could have been determined for intact tea bags, which is an essential improvement over the destructive methods of analysis. State-of-the-art in this area is exemplified, e.g., in the study on herbal tea blends (*Sceletium tortuosum* and *Cyclopia genistoides*) by Sandasi et al. (2018). The authors acquired hyperspectral images of the raw material and tea blends in 10870-3978 cm^-1^ (920-2514 nm) region. The images were subsequently analyzed with PCA, which revealed a distinct (54.2%) chemical variation between the raw materials. Next, a partial least squares-discriminant analysis (PLS-DA) model was constructed with prediction power of 95.8%. Applied for pixel classification, it enabled to visualize the distribution of the constituents *S. tortuosum* and *C. genistoides* and quantitatively predict their contents within the blend tea samples. It was pointed out that HSI instrumentation with a better sensitivity could potentially further improve the quantitative analysis in this and similar cases.

Kava (*Piper methysticum*) is a widely popularized pharmaceutical product containing root extracts. However, kava roots may contain toxins that led to several reported cases of liver intoxication. In consequence, several countries banned import of kava materials and products in the past, and strict quality control procedures were introduced afterwards. The existing methods for this purpose are lacking in high-throughput capacity, and therefore, efforts are made to develop new, more capable strategies. Segone et al. (2020) adopted NIR-HSI system operating in 10870-3978 cm^-1^ (920-2514 nm) region for rapid assessment of six kavalactone biomarkers (methysticin, dihydromethysticin, yangonin, desmethoxyyangonin, dihydrokavain, and flavokawains A and B) content in kava extracts (roots, peeled stems, and stump peelings). The feasibility study was supported by single-point NIR and MIR spectroscopy, as well as HPLC as the reference analytical method. One of the methods’ intended purposes was to reliably discriminate kava roots from non-roots samples. PCA algorithm was used to identify spectral variance correlated best with the chemical differences between these two types of sample. The compounds identified as the primarily responsible for the chemical differences between the plant parts were kavain, methysticin, and yangonin. NIR-HSI technqiue achieved good classification performance, with the prediction accuracy extending beyond 90%. The developed approach was reported to be suitable for automation and for continuous operation, fully suitable for practical application as a high-throughput quality control tool.

As the result of imperfect storage conditions, mycotoxigenic fungi may appear in foodstuff produced from plants, e.g., cereals. A significant health risk may result from intoxication by mycotoxins contributed by these plant pathogens. The potential of NIR-HSI technique in the role of the detection tool for mycotoxins in cereals was recently reviewed by Femenias et al., 2020. As summarized, the analytical performance of HSI approach is decisively superior to both the conventional methods of wet analytical chemistry and multispectral imaging methods. The capacity for high-throughput cereal sorting combined with quantitative analysis of mycotoxin content (e.g., deoxynivalenol, DON), in the scenario where visual assessment is far inferior, were emphasized as the most significant advantages of NIR-HSI technique used in this role. Nonetheless, Femenias et al. (2020) pointed out that certain limitations, e.g., prediction accuracy and limit of detection, need to be further improved in order to establish this technique as an industry standard.

Conventional methods, often human classification, are the standard routes in the industry for the selection of tobacco leaves according to their quality. Marcelo et al. (2019) demonstrated how application of NIR-HSI and chemometric tools could improve discriminant analysis of tobacco leaves. Imaging data collected for standard tobacco leaf bundles was subjected to rapid MVA classification by means of support vector machine-discriminant analysis (SVM-DA) within 5 s in real time. The developed classification models accurately predicted tobacco’s chemical properties important for its quality characteristics in a more robust and reliable way, without subjectivity or bias of a human classifier.

Noteworthy, reliable and objective tools for detection and identification of *Cannabis sativa* are essential for uncovering illegal cannabis plantations. Pereira et al. (2020) developed a method based on NIR-HSI hyphenated to MVA algorithms (sparse PCA and soft independent modelling of class analogies, SIMCA) for this purpose. The method achieved sensitivity and specificity levels of 89.45% and 97.60%, in the case where evaluated samples included leaves of cannabis and similar plants and only 4 spectral variables (spectra points) were required for a sparse SIMCA model. That study evidenced a sufficient classification performance of NIR-HSI technique even when just four spectral bands are captured by the sensor, making it feasible to be implemented in low-cost airborne devices.

#### Optimization of the Cultivation Conditions

Point-spectroscopy accomplishes significant successes in monitoring the quality parameters of plants and finds use in practice, e.g., for the determination of the optimal harvest time of medicinal plants, which has strong correlation with the content of the bio-active compound in the plant and thus its therapeutic value. For example, Pezzei et al. (2017) in their trend-setting study applied novel miniaturized NIR spectrometer, which is laboratory independent and can operate directly in-the-field, to non-invasively examine common vervain (*Verbena officinalis*). Additionally, the analytical performance of the portable sensor towards quantitative analysis of the key constituents (verbenalin and verbascoside) was compared using a benchtop spectrometer as the spectroscopic reference method. The study revealed that NIR vibrational spectroscopy shows full feasibility for direct measurements of the pharmaceutical active ingredient (PAI) in fresh plant material and enables straightforward determination of the ideal harvest time of a medicinal plant. It is expected, that with the emerging portable HSI systems, the current envelope will be pushed forward, with the new possibility to extend the information presently available from in-the-field spectroscopy (i.e., chemical composition, PAI content, quality parameters, growth conditions) by adding the ability to unveil spatial distribution of that information in a fresh plant.

Noteworthy, quality of wheat crops expressed by the nitrogen nutrition index NNI (Bowling et al., 1980) may be monitored over wide agricultural areas using novel NIR-HSI systems mounted on unmanned aerial vehicles (UAVs). This high-throughput data can be used with high efficiency for determining the optimal cultivation conditions, e.g., irrigation strategy. Remote sensing approaches advance rapidly and become capable of quantitative analysis. Liu et al. (2020) employed remote sensing setup with flight altitude of 50 m that operated over Vis/SW-NIR region (22,222–10,526 cm^-1^; 450–950 nm) and spectral resolution of 4 nm to perform quantitative determination of NNI in winter wheat. For reference, the study additionally involved a ground-based NIR-HSI instrumentation with nominally better performance; wide spectral region of 28,571–4,000 cm^-1^ (350–2,500 nm) and maximum spectral resolution of 1 nm. The study showed that varying NNI in wheat yields a clear and distinct footprint in Vis/SW-NIR images, and the spectral resolution is a less critical factor here. The significant and highly characteristic response to NNI was observed in the green and red wavebands was observed. Further, quantitative models of NNI were successfully established and the accuracy of UAV-based imaging was fully satisfactory when compared with the performance achieved by ground-based benchtop instrument. The results collected from wide area monitoring by remote sensing HSI yielded highly useful practical information leading to improvements in the strategy for crop irrigation, to which the optimal approach changes depending on the growth stage of wheat. Rapid progress is made in the instrumentation and data analytical tools used for remote sensing HSI, with satellite-based HSI systems operating in NIR region (e.g., NASA’s Surface Biology and Geology mission, Indian Hyperspectral Imaging Satellite) intended for global-wide monitoring of agricultural crops (Aneece and Thenkabail, 2018).

#### Phenotyping

Various imaging techniques attract current attention as the tools for phenotyping. This topic in a broader context was reviewed recently by Biswas et al. (2020). Special attention should be given here to HSI approaches based on SW-NIR and NIR spectroscopy, owing to particular practical advantages of the technique and instrumentation (*The Advantages and Limitations of Spectral Imaging for Examination of Plant Tissues, Products and Related Materials* and *Simultaneous Applications of Different Spectral Imaging Techniques*). Bodner et al. (2018) proposed a novel approach based on NIR-HSI for characterization of the root system architecture and its functional role in resource acquisition in soil grown plants. The imaging instrumentation operating in the spectral region of 10,000–5,882 cm^-1^ (1,000–1,700 nm) with a spatial resolution of 0.1 mm and 222 narrow detector channels was employed to scan root systems of durum wheat (*Triticum durum*) grown in soil-filled rhizoboxes. In contrast to the more conventional root phenotyping by multispectral RGB imaging limited to assessing color contrasts between roots and growth media or artificial backgrounds, an imaging technique based on NIR spectral signatures largely improves the quality of information elucidated from the sample, including insights into chemical constituents and physico-chemical properties of roots and soil. To provide phenotyping capability, high-throughput data-analytical tools were optimized for a high degree of automation in image processing. Chemometric analysis with use of unsupervised clustering and thresholding approaches was critical to treat image segmentation and to enhance the effective spatial resolution. This unraveled distinctive radial composition of root axes and their decomposition dynamics. Compared to the routinely used multispectral RGB imaging, the developed high-throughput NIR-HSI approach improved significantly the amount of chemical information and phenotyping capability, although at the cost of more complex and less rapid measurements.

Briglia et al. (2019) examined drought stressed grapevines (*Vitis vinifera*) with aim to establish whether spectral imaging may be used for affordable, non-destructive phenotyping based on information about morphophysiological traits (leaf area, plant water consumption, leaf water potential). NIR-HSI and multispectral (RGB) techniques were evaluated for a possible implementation. That study aimed to identify water-stress combining both morphometric (leaf area) and physiological (water consumption) responses under various drought levels. RGB and NIR imaging techniques were confirmed to be feasible solutions for implementation as affordable phenotyping tools, and a suitable basis for development of new tools for a precision irrigation. As reported, the achieved results are meaningful for future standardization of phenotyping protocols meeting the current goals issued by the global phenotyping community.

Efficient food production in salinized lands is one of the major challenges of modern world. Search for rapid and reliable tools capable of selecting crops tolerant of salinity leads to focus is an active field of research. Non-destructive techniques capable of high throughput plant phenotyping based on morphological and physiological treats are deemed essential for accelerating plant breeding processes in sustainable agriculture. Feng et al. (2020) adopted Vis/SW-NIR-HSI (26,315–9,259 cm^-1^; 380–1,030 nm) instrumentation to monitor the plant phenotypes of okra (*Abelmoschus esculentus*). Samples from the 13 okra genotypes were examined after 2 and 7 days of salt treatment. Novel approaches for image analysis based on deep learning enabled improved performance in segmentation of plant and leaf. Deleterious effects of salinity disturbing the physiological and biochemical processes in okra were manifested as substantial changes in the spectral information. Vis/SW-NIR-HSI combined with deep learning approach was reported to be highly capable tool for high‐throughput phenotyping under salinity stress conditions.

### Raman Imaging

#### Investigation of Plant Microstructure

Raman imaging offers high lateral resolution and confocal resolution, i.e., the ability to perform imaging at a controlled depth beneath the specimen surface. Raman imaging instrumentation based on the excitation laser emitting in NIR wavelength region has made possible investigations of green plant material by mitigating the autofluorescence in the sample. Structural studies on plant cell walls are established using 1064 nm laser-based Raman imaging (e.g., Agarwal, 2014). However, longer wavelength increases the diffraction-based limit of spatial resolution (*Basics of the Spectral Image Acquisition*). Hence, investigations into feasible application of Raman imaging that employs visible (Vis) laser for higher spatial resolution are important. Several studies showed that such systems could be successfully applied for examining micro-structure of plant specimens in their native state with no need for staining or complicated sample preparation. Therefore, this technique finds particular usefulness for exploring the structural complexity of plant organisms. The internal microstructure of plants is mostly defined at the molecular level by the cell walls. These consist of semi-crystalline cellulose fibrils of thickness in few μm range, which are embedded in an amorphous matrix constituted of biopolymers (pectins, hemicelluloses, and lignins). Structural arrangement of these constituents within the cell wall varies among different plant tissues; therefore, techniques capable of investigating the spatial inhomogeneity of this feature are essential. Recent review articles covering this topic should be noted (Durrant et al., 2019; Zhao et al., 2019; Prats Mateu et al., 2020).

Raman imaging operating at μm resolution has been established as a particularly potent tool for such purpose, as e.g., demonstrated by Gierlinger (2014) in her study of wood specimens. Two-dimensional Raman mapping with Vis (532 nm) excitation laser was performed for micro-cross-sections of spruce (softwood) and beech (hardwood). The imaging data was analyzed using both univariate (band integration, height ratios) and multivariate methods (vertex component analysis, VCA). It was concluded that MVA approach yielded superior information, as VCA algorithm successfully separated anatomical regions and cell wall layers according to the most different molecular structures. In comparison, univariate approach only visualized changes in selected band heights or areas with weaker correlation to the morphological features. With high spatial resolution (<1 μm), the investigation revealed subtle changes in lignin composition and content in the cell walls, which has been ascribed to a non-uniform lignification during growth of the specimens. Thus, detailed information on the inhomogeneous distribution of structural features of plant cell walls was derived. Alongside gains for basic plant science, these results had potential practical importance for optimizing the utilization of plant biomass (Gierlinger, 2014).

**Figure 8:**
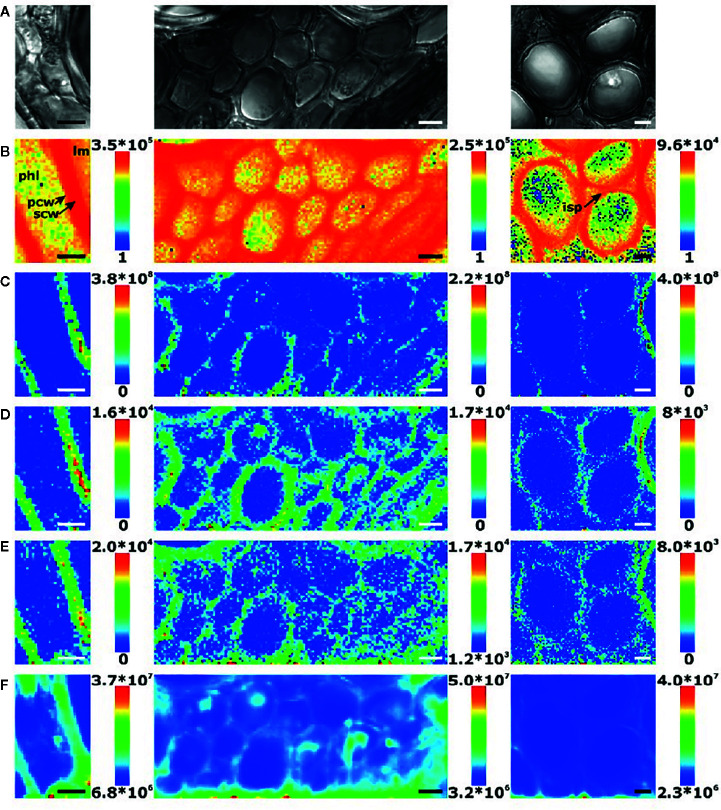
Raman chemical images of three exemplary mapping data sets of cross sections of cucumber (*Cucumis sativus*) stem xylem. **(A)** Bright field images; **(B)** Integral intensity in the region 1,550–1,700 cm^−1^, obtained after baseline correction; **(C)** Product of the intensities of the cellulose bands at 1,092 cm^−1^ and 1,337 cm^−1^, respectively, cf. **(D, E)**; **(D)** Intensity at 1,092 cm^−1^ (baseline corrected in the range 1070–1108 cm^−1^); **(E)** Intensity at 1337 cm^−1^ (baseline corrected in the range 1,313–1,358 cm^−1^); **(F)** Intensity integrated over the full spectral range 600–2,000 cm^−1^. lm, lumen; phl, phloem; scw, secondary cell wall, pcw: primary cell wall; isp, intercellular space. Scale bars: 10 μm, mapping step size: 1 μm, excitation wavelength: 532 nm, excitation intensity: 1.7 × 106 W/cm^2^, accumulation time: 1 s. Reproduced in compliance with CC-BY 4.0 license from Zeise et al., 2018.

The application of Raman imaging technique for examination of the chemical and structural properties of cell walls remains a particularly active and fruitful area of research (Durrant et al., 2019; Zhao et al., 2019; Prats Mateu et al., 2020). Recent investigation by Zeise et al. (2018) demonstrated how high-resolution spatial distribution of chemical composition available from Raman microspectroscopy can be used to gain deeper insights into the properties of microscopic sub-structures of the plant tissue, e.g., the building blocks of the cell walls. The study focused on cucumber (*Cucumis sativus*) with the experimental based on 55 Raman maps of root, stem, and leaf tissues. Through spectra analysis carried out with both univariate and multivariate algorithms, different spectral contributions from cellulose and lignin could be unraveled in Raman images. Contributions from the main cell wall components, lignin and cellulose, were suitable for assembling univariate (chemical) images of the sections and revealed substructures of the cell walls in the xylem tissue (**Figure 8**). Further, images constructed through MVA with hierarchical cluster analysis (HCA) and principal component analysis (PCA) algorithm identified different substructures in the xylem cell walls among different tissues and visualized the cell wall substructures more clearly. Noteworthy, the laser excitation wavelength range (532 nm) enhanced the signal from carotenoid species (at 1,523 cm^-1^, 1,156 cm^-1^, and 1,005 cm^-1^) through resonance effect. Zeise et al. (2018) presented different possible approaches of generating Raman images with high contrast and resolution suitable to analyze morphological information acquired from sections of native, unembedded root, stem, and leaf tissues of cucumber plants.

Raman imaging technique offers great potential for monitoring of structural and compositional changes at plant cellular level. With spatial resolution reaching down to ca. 300 nm, Raman imaging is capable of elucidating molecular structural features of plants at cellular level. For example, Gierlinger et al. (2012) conducted a trend-setting study on chemical imaging of plant cell walls at sub 0.5 μm level by confocal Raman microscopy. Embedding and microcutting procedures of sample preparation were developed with aim to preserve plant tissues with intact cell walls. Alongside, data-analytical approaches were designed to present the images and to resolve molecular fingerprints among the multiple components appearing within the native cell walls. The study provided insights into polymer composition as well as the orientation of the cellulose microfibrils. The potential of this technique includes the ability to follow changes occurring in the structure and chemical composition of plants at single cell level; these temporal features can be monitored within different cells as well as between them. Research and results obtained at this direction were summarized in detail by Prats Mateu et al. (2014).

Li et al. (2020) employed confocal Raman microscopy to investigate the distribution of water content in apple tissues at the cellular level with valuable insights into the water molecular state with respect to hydrogen bonding (HB). The spectra of fresh apple tissues featured five peaks in the region of 3,000–3,800 cm^−1^, that were assigned to the OH stretching mode of water molecules in different local environments resulting from the difference in the hydrogen bonding states. Interestingly, water molecules with the strongest and the weakest hydrogen bond (corresponding to the peaks at 3,050 and 3,630 cm^-1^) were identified to be primarily located in the cell wall areas.

This observation demonstrated the potential for elucidating useful information on the water migration in plant tissue as cellular level, as well as practical value for molecular effects of food processing, e.g., freezing or drying.

Raman imaging shed light on the physiological changes underlying wood degradation in the study by Belt et al. (2019). The investigation focused on identifying the key steps in heartwood degradation in the incipient stages of brown rot decay in scots pine (*Pinus sylvestris*). The study unveiled that the degradation of heartwood begins in the innermost cell wall layers and then spread into the remaining cell walls and the middle lamella. One of the most prominent of the observed Raman spectral changes were identified as the decrease in the intensity of the bands due to pinosylvins. Further, this enabled monitoring an extensive degradation of pinosylvins in the cell walls, middle lamella and extractive deposits were observed. Other changes were observed as well, leading to the conclusion that the key driver of the incipient heartwood decay is the degradation of antifungal heartwood extractives, accompanied by the degradation of the inner cell wall and introduction of degradative agents into the cell walls and middle lamella.

#### Characterization of Spatial Distribution of Carotenoids

Carotenoids are common pigments abundant in numerous plants and algae as well as bacteria and fungi. They play an important physiological role in plant biology, as they form a protective measure against photodamage from overexcitations and are critical in absorbing energy of incident radiation and thus are essential for photosynthesis. Raman spectroscopy and imaging was extensively used for investigations of carotenoids in plant tissues (Schulz et al., 2014; Pećinar, 2019). Oleszkiewicz et al. (2020) provided a detailed example of the sample preparations aimed to preserve intact tissue for subsequent analysis using Raman spectroscopy. Characteristic bands of several important carotenoids (e.g., β-carotene, lutein, and lycopene) are located at the following wavenumbers; *v*(C=C) peak at ca. 1535-1500 cm^-1^, *v*(C-C) at ca. 1,145–1,165 cm^-1^, *v*(C–CH_3_) at ca. 1,010–1,000 cm^-1^. These bands are sensitive markers of the molecular structure of a carotenoid, band shift deliver information on the number of conjugated bonds and the side groups in the molecule, as well as its interaction with the chemical environment (i.e., matrix). Carotenoids are uniquely meaningful for Raman imaging studies of plants because of the resonance-based enhancement of their *v*(C=C) signal in Raman spectra. This gives the possibility to distinguish between the distributions of different carotenoids in Raman images. The study by Schulz et al. (2005) may serve here a classical example in which Raman imaging could be successfully used to extract individual distribution of carotenoids with 7, 8, and 9 conjugated bonds from the intact tissues of pot marigold (*Calendula officinalis*). As another example, β-carotene distribution in sweet potato, carrot and mango was examined by Brackmann et al. (2011) using imaging system based on coherent anti Stokes Raman scattering (CARS) principle. Sensitive probing of the β-carotene was achieved by monitoring its characteristic signal at 1,520 cm^-1^. This enabled unraveling different macro-structural assemblies in each species. Sweet potato and carrot features rather densely accumulated β-carotene forming heterogeneous rod shaped structures, while homogeneous aggregates of carotenoid filled lipid droplets were identified in mango.

High-resolution Raman imaging can be used for highly detailed *in situ* characterization of carotenoid properties, as demonstrated by Roman et al. (2015). The conclusions drawn from that study shed light on the distribution and composition of crystalline and amorphous carotenoids in carrot cells. Crystalline carotenoid domains contain α-carotene, although the presence of lutein could not be excluded. In contrast, amorphous carotenoids are composed of β-carotene molecules. Additionally, through the band shifts observed in Raman spectra, it was concluded that amorphous carotenoids are involved in intermolecular interactions with other plant constituents, e.g., proteins or lipids. These accomplishments displayed the differences present in the carotenoid content in carrot with respect to crystalline and amorphous state. When combining Raman imaging with other techniques, further insights into the structure and properties of crystalline phase carotenoids in carrot root can be elucidated, as shown by Rygula and associates (2018). For the first time, that study unveiled the chemical and structural differences of carotenoid crystals, including the varying composition of the crystals. Evidence was presented on the uniform chemical composition of the crystals, regardless of their planar structure. The exception was observed for the helical crystals, where α-carotene was deemed absent.

Carotenoids play a role in the biochemical adaptive protection mechanisms of certain plants. Algae serve as highly suitable model plant organisms and are important part of the ecosystem. Therefore, they attract considerable attention in plant science (Coman and Leopold, 2017). Similar to various other algae, upon light irradiation *Chlorella* species (*C. protothecoides* and *C. vulgaris*) produce and accumulate large amount of carotenoids within their cells. This phenomenon was investigated by Grudzinski et al. (2016) using Raman mapping technique. It was found that light induced yellowing of *Chlorella* sp. results from the accumulation of xanthophylls, primarily zeaxanthin. It was accomplished at good sensitivity and selectivity levels, through arranging selective Raman resonance conditions using two spectral acquisitions with 488 and at 514 nm excitation lasers. At both of these wavelengths a resonance condition for xanthophyll pigments is achieved, while for zeaxanthin this occurs only for 514 nm line. It was revealed that under strong light exposure conditions carotenoids are formed at cell nucleus, with additional qualitative information about zeaxanthin being the major synthesized carotenoid. This study revealed an adaptive mechanism of plant against intense radiation in visible region, referred by the authors as ‘the molecular sunglasses’ presumably intended to shield the sensitive cell nuclei. These conclusions brought important insights into the adaptive mechanisms of algae against overexcitation. Further, practical gains from this discovery is the potential cultivation of *Chlorella* for use of as the alternative source of zeaxanthin, as it a macular pigment suiting protective role in human eyes against the age related macular degeneration (Grudzinski et al., 2016).

Noteworthy, alongside the major constituents, Raman imaging is capable of elucidating the distribution of other class of compounds within plant’s body (Coman and Leopold, 2017). Polyacetylenes can be monitored at ca. 2100-2300 cm^-1^ through the *v*(C=C) vibration. For instance, Barańska et al. (2005) studied the distributions of falcarinol and falcarindiol in carrot and concluded that their accumulation occurs primarily in the outer sections of the roots.

Raman mapping was employed by Sharma et al. (2015) for unraveling the localization of major types of biomolecules within the cells of *Chlamydomonas reinhardtii* microalgae. The distribution of proteins, lipds and carotenoids was performed *via* their characteristic wavenumbers, at 1,003, 1,445, and 1,520 cm^-1^, respectively. The generated Raman images highlighted the lipid rich, carotenoid rich and protein rich areas in the cells. Further, characterization of lipids enabled identifying among the mutated cells those with the increased lipid content.

### Fluorescence Imaging

As outlined in Introduction, the aim of the present section is to briefly overview the most recent applications of fluorescence imaging, and on this background, to better present the potential of vibrational spectral imaging methods in plant science. CFI technique has found significant use in investigation of the influence of biotic stress delivered to living plants in various ways. Photosynthesis, as a metabolic process critical for plant physiology, is distinctly affected by external factors, e.g., exposition to pathogens and environmental aspects (Pérez-Bueno et al., 2016). This includes manipulations of the plant metabolism induced by the pests, but also plant’s own defense mechanisms, in which photosynthesis rate is reduced in order to limit the availability of nutrients to the pathogens. Regardless of the cause, biotic stress typically causes spatially and temporarily heterogeneous effects, which can be elucidated by CFI through monitoring photosynthetic activity at cellular level.

**Figure 9:**
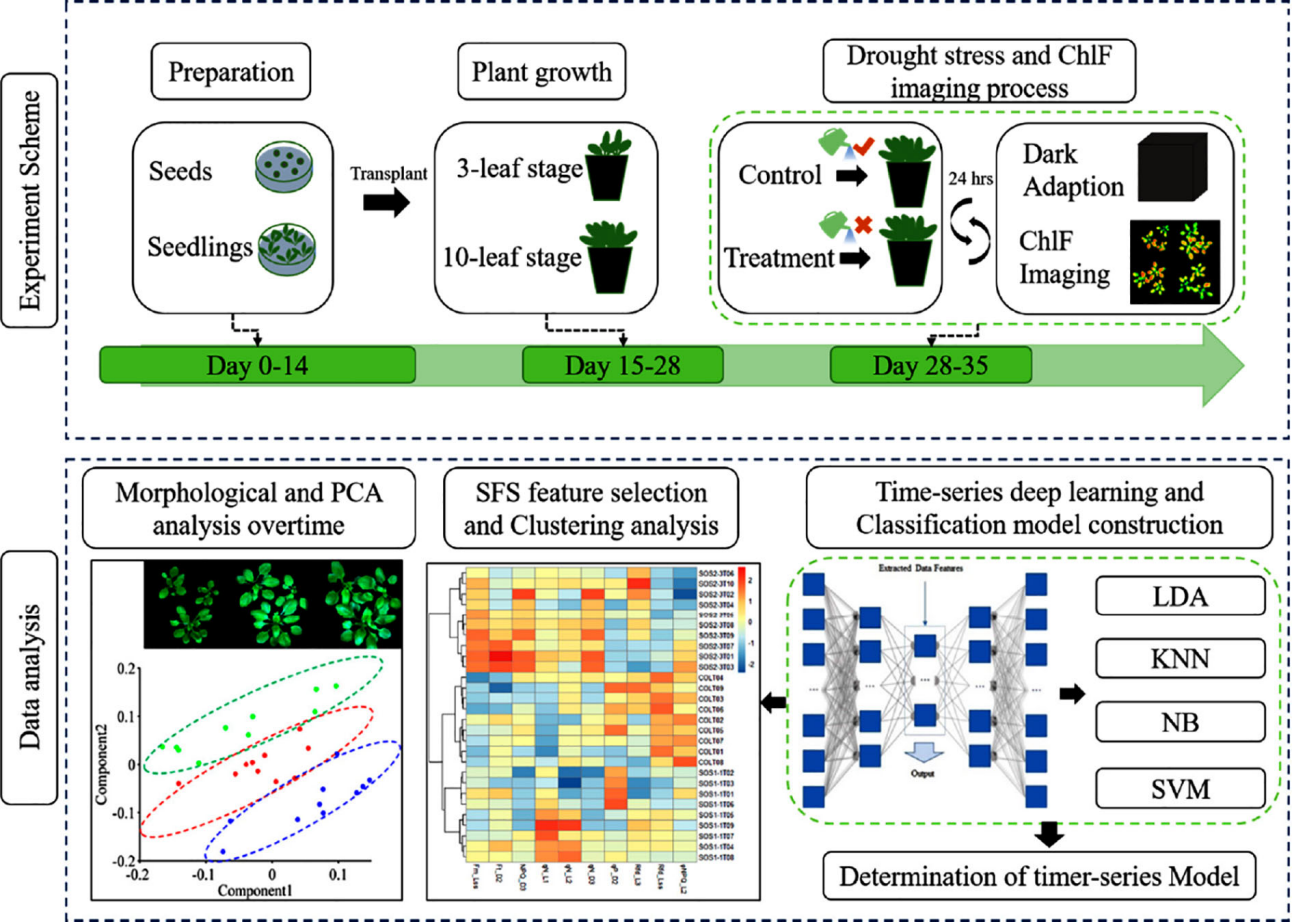
Workflow of using chlorophyll fluorescence imaging (CFI) technique to dynamically monitor photosynthetic fingerprints caused by salt overly sensitive genes under drought condition. LDA, linear discriminant analysis; KNN, k-nearest neighbor classifier; NB, Gaussian naive Bayes; SVM, support vector machine. Reproduced in compliance with CC-BY 4.0 license from Sun D. et al. (2019).

Drought and salt stress are extremely meaningful sources of biotic stress in plants, and their primary significance for public stems from the impact they have on agriculture. These conditions perturb the functional properties of photosystem II (PSII) and lower the amount of energy available from the process, to which plants respond by reducing biochemical activities (Zhu, 2002). Further side-effects may be noted, including oxidative stress, osmotic stress, increased ion toxicity and disturbed homeostasis of Na+ and K+ cations (Zhu, 2002; Sun D. et al., 2019). Therefore, detailed knowledge on the metabolic effects these factors induce on agricultural plants is essential for the attempts to improve breeding programs towards better resilience to drought. CFI techniques can form extremely helpful tool in such research, as demonstrated, e.g., in a recent study by Sun D. et al. (2019). They examined the behavior of salt overly sensitive (SOS) mutants of thale cress (*Arabidopsis thaliana*) as a model plant using a time-series CFI analysis to dissect the chlorophyll fingerprints of salt overly sensitive (SOS) mutants under drought conditions. The investigation employed a potent set of data-analytical tools to unravel the chlorophyll fingerprint and its patterned change under drought and salt stress conditions. Authors employed PCA to resolve the shifting pattern of different genotypes in the examined specimen including SOS mutants and wild type plants. Subsequently, temporal features were elucidated using sparse auto encoders (SAEs) neural network, a time-series deep-learning algorithm. The resulting data were used in chemometric classifications based on linear discriminant analysis (LDA), k-nearest neighbor classifier (KNN), Gaussian naive Bayes (NB) and support vector machine (SVM), with very good accuracy of discrimination between the specimen. In addition, the authors employed sequential forward selection (SFS) algorithm was used to identify the most characteristic chlorophyll over-time responses to drought stress of each specimen. The complex workflow designed by for their study is presented in **Figure 9**. The accomplished results demonstrate the suitability of CFI approach supported by sophisticated data-analytical procedures, to monitor the gene function underlying plant’s physiological response to biotic stress induced by drought and increased saltiness of the environment (Sun D. et al., 2019).

Noteworthy are selected earlier developments, e.g., those aimed at improving deep-tissue capability of plant imaging. Conventional approaches to this problem require problematic sample preparations, as explained in *The Advantages and Limitations of Spectral Imaging for Examination of Plant Tissues, Products and Related Materials*. Warner et al. (2014) developed an improved method with aim to simplify deep-tissue imaging by fluorescence and make it suitable for examining intact plant organs and whole plants. The method was based on an alternative approach to chemical cleaning intended for greater tissue transparency and light transmission while preserving the ability to use common fluorescent stains and proteins. The procedure was based on clearing solutions with lowered intrusiveness, which lead to better preservation of subcellular features, enhance light transmission through the sample, and retain the ability to use common fluorescent stains and proteins. Warner et al. (2014) reported that their approach enabled successful imaging of fine cellular features in specimens, while maintaining the refractive indices of cleared plant tissues at the close level to that of untreated specimen.

Considerable attention should be given to the emerging applications of CFI as a part of integrated remote sensing techniques with potential to advance modern precision agriculture. In such applications, the pursued goals are to monitor large areas of crop fields in combination with the immense capabilities of spectral imaging methods. Unlike the typical applications of CFI, mostly intended to examine plant defense responses to stress factors, novel concepts focus on the ability to perform plant phenotyping, e.g., as reported by Pérez-Bueno et al. (2016). That study investigated the feasibility of applying multicolor CFI (MCFI) in combination with thermography for remote sensing and phenotyping purposes. Such abilities depend on processing large and complex data sets, and the study by Pérez-Bueno et al. (2016) included development of data-analytical methods valid for intended tasks. In the process, a number of statistical models were evaluated, including logistic regression analysis (LRA) and artificial neural networks (ANN). These models have subsequently been validated in real life scenario and demonstrated the performance comparable to the established conventional techniques; however, the developed method of MCFI in combination with thermography is superior in scalability.

CFI has been demonstrated to be a potent tool for monitoring the damage induced to plants by environmental pollution by heavy metals. The process of photosynthesis appears to be particularly sensitive to appearance of heavy elements, cadmium in particular. Bayçu et al. (2018) conducted a CFI-based study to provide new data on the mechanism of plant acclimation to exposure to Cd element (Parmar et al., 2013). They examined how the PSII of *Noccaea caerulescens* behaves during plant acclimation to the exposure conditions. CFI technique enabled a non-invasive visualization of the spatiotemporal variations in PSII efficiency and offered insights into the mechanism of PSII acclimation to Cd exposure. Further insights into cadmium effects could have been obtained by Moustakas et al. (2019) by combining CFI with a technique of laser ablation inductively coupled plasma mass spectrometry (LA-ICP-MS). With aim to unravel the impact of Cd accumulation on the plant’s PSII photochemistry, they examined clary sage (*Salvia sclarea*) with focus on following the decrease of its photosynthetic efficiency as a result of Cd exposure. CFI and LA-ICP-MS could be successfully combined to monitor heavy metal effects and plant tolerance and the mechanisms the plants develop for Cd detoxification. CFI enabled determining the spatial distribution of the heterogeneous changes occurring in the plant leaf. The study revealed that *Salvia sclarea* tends to accumulated Cd and exhibits good tolerance level to the presence of this element and may be used in the role of a phytoremediant plant (Moustakas et al., 2019).

### Simultaneous Applications of Different Spectral Imaging Techniques

**Figure 10:**
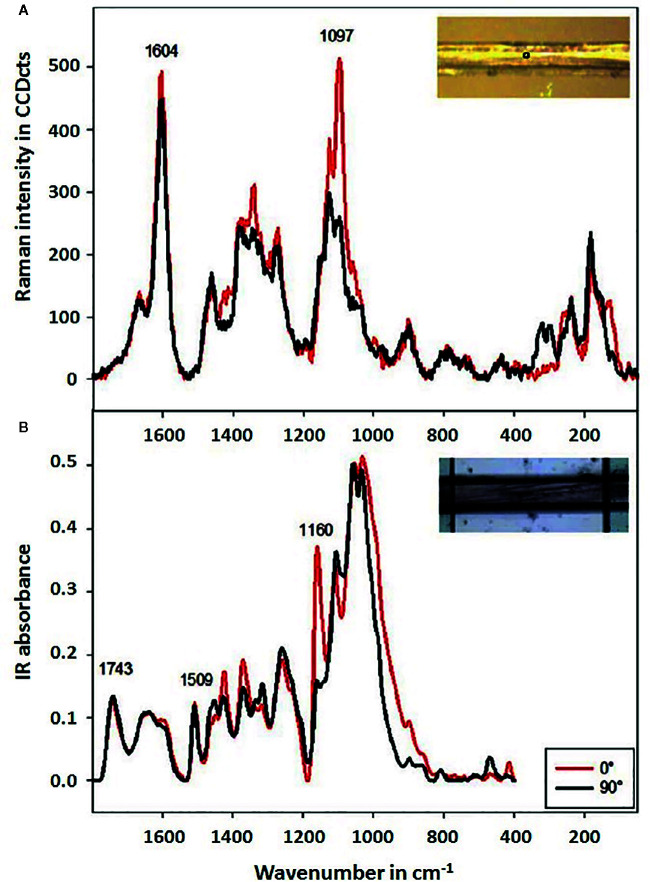
Baseline corrected Raman **(A)** and Infrared spectra **(B)** acquired on mechanically isolated single spruce wood fibers (microfibril angle < 10°) with the incident polarization direction parallel to the fiber (0°, red spectra) and perpendicular to the fiber (90°, black spectra). Reproduced in compliance with CC BY-NC-ND 4.0 license from Gierlinger, 2018.

It may be noted, that the imaging instrumentation and spectra processing tools achieved a reasonable level of maturity nowadays. Further progress likely depends on improving the ability to interpret complex chemical signatures typically encountered in plant samples. In order to improve the chemical specificity and interpretability of spectral images, applications of complementary techniques is highly promising. For the reasons explained in *Basic Information Related to Spectra Origin and Interpretation*, a good combination is formed by IR and Raman imaging, as correlations between the spectral features can mutually aid interpretation of both types of imaging data. As demonstrated by Gierlinger (2018), interrogation of a plant specimen by FT-IR and Raman confocal microscopy leads to a deeper understanding of biological processes and structure–function relationships occurring therein. The combined techniques largely increase the chemical specificity and interpretability of the acquired images (**Figure 10**) offering better understanding of critical factors, e.g., plant cell wall chemistry or biochemical functions of microstructures. The complementary character of IR and Raman imaging techniques in the context of plant science has been well summarized by Gierlinger (2018). It is our opinion, that NIR spectroscopy adds significantly to this combined potential, particularly owing to significant progress that is being currently achieved towards interpretability of NIR spectra. The synergy between the imaging techniques based on different modalities of vibrational spectroscopy is presented in **Table 4**. At the same time, MVA techniques such as unmixing methods (e.g., vertex components analysis) are helpful in addressing overlapping bands, which are common in the spectra of biochemically complex samples. Recent progress in developing unmixing algorithms with the purpose of improving the interpretability of Raman imaging of plant cell walls is noticeable (e.g., Prats Mateu et al., 2018). Over the current decade, one may anticipate increased attention paid to improving our understanding and ability to interpret the chemical information entangled in spectral images acquired from plant specimens.

**Table 4:** Comparison of the principal characteristics of near-infrared (NIR), infrared (IR), and Raman microspectroscopy relevant to imaging studies in plant science.

	**NIR**	**IR**	**Raman**
**Excitation mechanism**	Absorbance	Absorbance	Scattering
**Complexity of instrumentation**	Low	Medium	High
**Selection rule (chemical sensitivity)**	Change in dipole moment (polar moieties, enhanced signal of X-H groups, e.g., O-H, N-H, C-H)	Change in dipole moment (polar moieties)	change in polarizability (non-polar symmetrical bonds, e.g., C–C, skeletal vibrations)
**Confocal resolution/in-depth sampling**	Not possible	Not possible	~0.6–4 μm
**Sampling (i.e., spectra acquisition modes)**	Transmission; diffuse reflection; transflection	Transmission; reflection; transflection; cassegrain or attenuated total reflectance (ATR)	Reflection (scattering); immersion microscope objectives with high numerical aperture possible
**Remarks about sample preparation**	No/minimal sample preparation needed; Moderate suitability of water as solvent or glass as container/optics	In transmission mode: optimal sample thickness; In ATR mode: optimal/stable sample-IRE contact surface;	Plane surface for mapping/imaging; no contact mode necessary; suitability of water as solvent or glass as container/optics
**Chemical specificity**	Low to moderate	High	High
**Major issues and challenges**	Low sensitivity; Overlapping contributions in the spectra; Difficult spectra interpretation;	Limited suitability of moist samples; Unsuitability of glass optics and materials (absorption of glass); Interfering signal from atmospheric H_2_O and CO_2_	Raman signal obscured by autofluorescence (stronger for excitation lasers with shorter emission wavelengths); Laser heating, danger of destruction of molecular structure, e.g., proteins; or sample thermal decomposition (particularly of dried material)

Information on the latter two techniques adapted in compliance with CC BY-NC-ND 4.0 license from Gierlinger, 2018.

**Figure 11:**
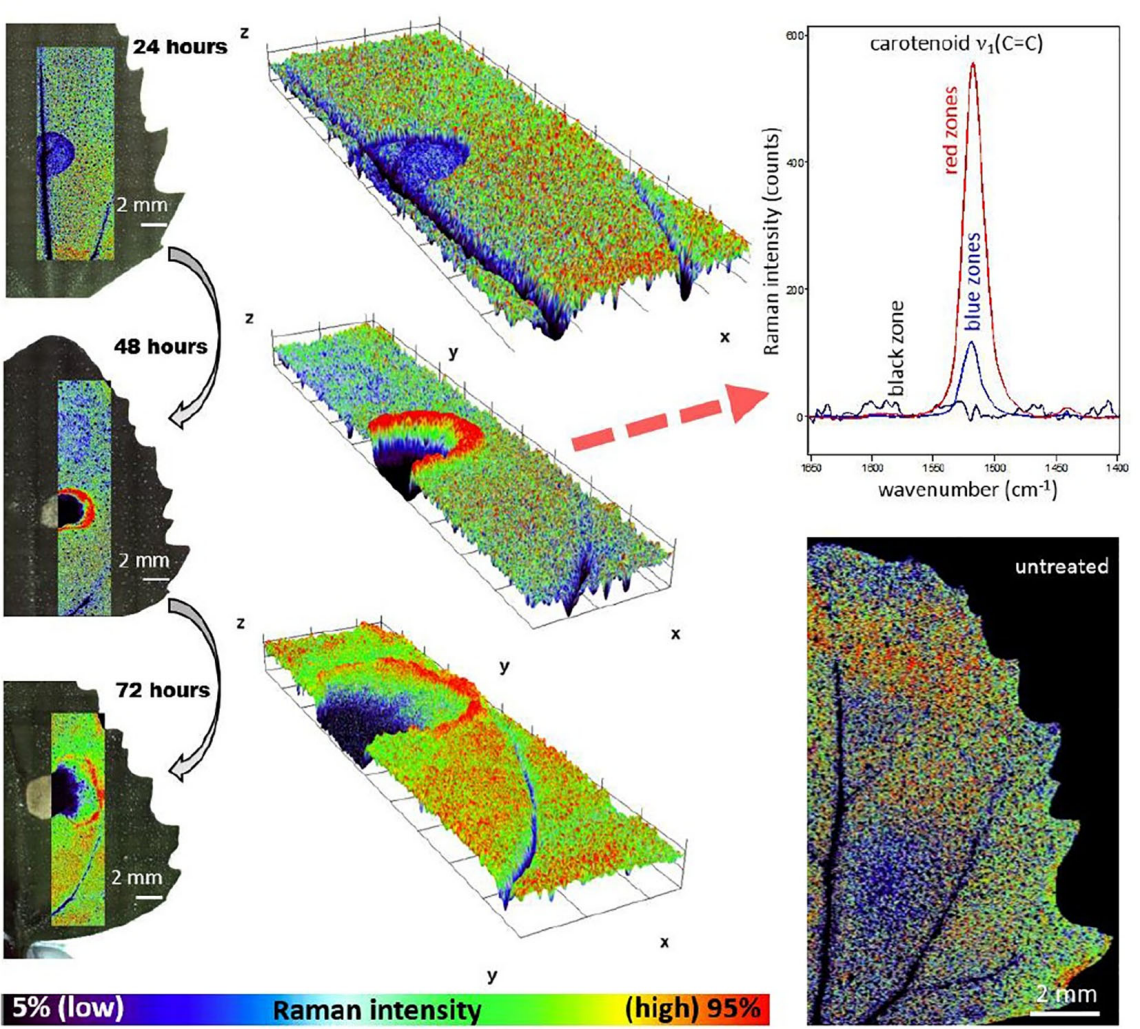
Distribution of Raman signal-to-baseline intensity of the carotenoid *v*_1_(C=C) band in leaves of *Chenopodium album* as unveiled by Vítek et al., 2020. The area around the herbicide application was scanned 1–3 days after application. The 3D plots represent a visualization of 2D spatial information, with *z*-axis representing the Raman intensity of the *v*_1_(C=C) band. Reproduced in compliance with CC-BY 4.0 license from Vítek et al., 2020.

**Figure 12:**
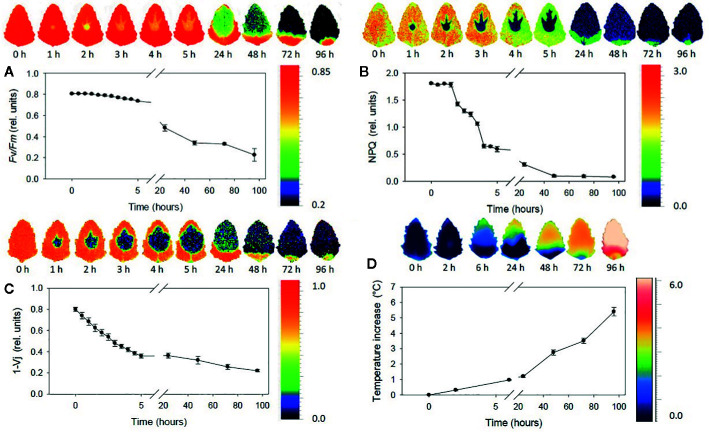
Sequence of chlorophyll fluorescence and thermal imaging of leaves in the span 0–96 h after application as unraveled by Vítek et al., 2020. **(A)** The maximum quantum efficiency of PSII (*F*_v_/*F*_m_). **(B)** Non-photochemical quenching (NPQ). **(C)** Probability that a trapped exciton moves an electron into the electron transport chain beyond primary quinone electron acceptors of PSII (1-*V*_j_). **(D)** Temperature increase in comparison to untreated control. Points represent means and error bars standard deviations (*n*=5). Reproduced in compliance with CC-BY 4.0 license from Vítek et al., 2020.

Worth emphasizing are investigations that combine vibrational and fluorescence spectral imaging to elucidate even more complete information from the sample. Recently, a trend-setting investigation was reported by Vítek et al. (2020). In this study, the authors combined different imaging techniques (Raman, CFI and IR thermal) to resolve the spatial and temporal changes that occurs as a plant’s response to PSII inhibition. A herbicide metribuzin was used as the inhibitor of the PSII in a model plant species *Chenopodium album*. High-resolution Raman imaging unveiled zones of local increase of carotenoid following the application of herbicide. The presence of carotenoids was highly correlated with the damaged tissue over time, as a result of the activation of defense mechanisms (**Figure 11**). Further, the Raman shift in the carotenoid band was a clear marker of the structural changes in carotenoids. CFI technique was employed to elucidate the spatial- and time-dependent variations in the quantum efficiency of PSII (**Figure 12**). In particular, it was possible to observe the spatial distribution of the key parameters of photosynthesis (*F*_v_/*F*_m_ and NPQ) and to unveil that the movement of herbicide acropetally (in direction to the leaf tip) mostly through main veins occurs within hours from the application of metribuzin. CFI demonstrated that the herbicide affects sharply defined parts with no transition areas. IR thermal imaging enabled observing patterned changes of leaf temperature induced by the herbicide (**Figure 12**), in relation to control specimen untreated with metribuzin, with the temperature elevating from 0 to above 5°C during the period 96 h after the application of the agent. The observations based on IR thermal imaging remained in agreement with those made in the CFI experiment; the temperature increase was relatively greater towards the upper part of leaf, confirming the acropetal movement of the herbicide. Noteworthy, this technique was concluded to be inferior to chemical imaging, as the herbicide transport through veins could not be monitored, neither the areas affected by the agent were as well-defined.

## Summary and Future Prospects

The present state-of-the-art plant science takes advantage of spectral imaging technique across numerous research directions. IR techniques benefit from chemical specificity and are well-suited to characterize molecular properties and spatial distribution of chemical contents, unveil microstructural features and follow biochemical processes ongoing in plants. NIR imaging does not require sample preparations, is more suitable for studies of moist samples and can be used to study water distribution in roots and soil. Because of deeper penetration of NIR light into the sample, this approach is more feasible to determine properties over larger volume in typically inhomogeneous plant material. This makes it preferable for applications where local variations of chemical compositions are not relevant or even detrimental, as it is in determinations of the total chemical contents, e.g., of pharmaceutically active ingredients in medicinal plants. For similar reasons, assessments of nutritional values, micro- and macro-nutrients concentrations and distributions, as well as other material quality parameters (e.g., relevant in agro/food applications), are easily obtained with NIR imaging. Further, deep-tissue sensing is possible in non-destructive manner. Direct interpretation of NIR spectra is difficult, although recent advances are promising. Raman imaging is a potent technique as it achieves superb spatial resolution, which makes it suitable for microstructure investigations and sub-cellular studies. Furthermore, it enables practical confocal resolution, enabling acquisition of information from beneath sample surface in a controlled manner. Care needs to be taken when examining specimens sensitive to thermal decomposition; recently a comprehensive dissection of this issue was published (Hauswald et al., 2019). This technique is fully suitable to study moist samples, although green plant material may pose a challenge because of interfering fluorescence signal. This feature may reduce the selectivity of Raman imaging, but still selected compounds like carotenoids can be easily elucidated. The characteristics sketched above should not be considered in absolute categories, as the capabilities of the reviewed techniques overlap to some extent. Additionally, simultaneous application of several imaging techniques can mutually mitigate their limitations and further elevate the abundance of information elucidated from the sample. Combined approaches seem to be an increasingly commenced trend with promising outlook. Alongside, imaging instrumentation is continuously improving. Developments stimulated by research goals set up in other fields, such as biomedical spectral imaging, are adopted into plant investigations; for example, high-performing synchrotron IR imaging. This progress is accompanied by the development of data-analytical methods and image generation algorithms.

## Author Contributions

KB designed and prepared the manuscript. KB and JG performed literature search. KB and JG edited the manuscript. CH supervised the entire process. All authors contributed to the article and approved the submitted version.

## Funding

This work was supported by the Austrian Science Fund (FWF): M2729-N28.

## Conflict of Interest

The authors declare that the research was conducted in the absence of any commercial or financial relationships that could be construed as a potential conflict of interest.
